# Ellipsoid Segmentation Model for Analyzing Light-Attenuated 3D Confocal Image Stacks of Fluorescent Multi-Cellular Spheroids

**DOI:** 10.1371/journal.pone.0156942

**Published:** 2016-06-15

**Authors:** Michaël Barbier, Steffen Jaensch, Frans Cornelissen, Suzana Vidic, Kjersti Gjerde, Ronald de Hoogt, Ralph Graeser, Emmanuel Gustin, Yolanda T. Chong, IMI PREDECT Consortium

**Affiliations:** 1 Discovery Sciences, Janssen Pharmaceutical companies of Johnson & Johnson, Beerse, Belgium; 2 Pharma R&D IT, Janssen Pharmaceutical companies of Johnson & Johnson, Beerse, Belgium; 3 Department of Urology, Erasmus MC Rotterdam, Rotterdam, The Netherlands; 4 Faculty of Mathematics, Natural Sciences and Information Technologies, University of Primorska, Koper, Slovenia; 5 Translational Medicine and Clinical Pharmacology, Boehringer Ingelheim, Ingelheim am Rhein, Germany; Pennsylvania State Hershey College of Medicine, UNITED STATES

## Abstract

In oncology, two-dimensional in-vitro culture models are the standard test beds for the discovery and development of cancer treatments, but in the last decades, evidence emerged that such models have low predictive value for clinical efficacy. Therefore they are increasingly complemented by more physiologically relevant 3D models, such as spheroid micro-tumor cultures. If suitable fluorescent labels are applied, confocal 3D image stacks can characterize the structure of such volumetric cultures and, for example, cell proliferation. However, several issues hamper accurate analysis. In particular, signal attenuation within the tissue of the spheroids prevents the acquisition of a complete image for spheroids over 100 micrometers in diameter. And quantitative analysis of large 3D image data sets is challenging, creating a need for methods which can be applied to large-scale experiments and account for impeding factors. We present a robust, computationally inexpensive 2.5D method for the segmentation of spheroid cultures and for counting proliferating cells within them. The spheroids are assumed to be approximately ellipsoid in shape. They are identified from information present in the Maximum Intensity Projection (MIP) and the corresponding height view, also known as Z-buffer. It alerts the user when potential bias-introducing factors cannot be compensated for and includes a compensation for signal attenuation.

## Introduction

Drug discovery and development in oncology has a success rate as low as 6% [1]. The traditional models used for oncology drug testing are monolayer cultures of tumor cells grown on glass and plastic substrates. These models are very different from solid tumor behavior in vivo. In comparison, 3D cultures are thought to more closely mimic the tumor microenvironment [2], as they allow more complex interactions between cancer and stromal cells, and expose cells to more realistic mechanic forces. Studies have demonstrated important differences in transcription profiles [3] and drug sensitivity [4–8] between 2D and 3D cell culture systems. For a recent review, see [9]. The PREDECT consortium aims to validate various 3D cell culture models by comparing their histology and protein expression with that found in patient samples [10].

An important category of such models are spheroid microtumors grown in a matrix, such as Matrigel^®^, with or without the presence of supporting stromal cells. Typically, cancer cells are seeded in the extracellular matrix in each microtiterplate well, and over time multiple spheroids of different sizes develop. These 3D multi-cellular spheroid models reflect more closely the nutrient, oxygen and drug gradients that can be found in a tumor, reproducing hypoxic, proliferative, apoptotic and necrotic regions [11, 12]. Suitable dyes or antibodies may be applied to detect these regions/processes, but an accurate quantification of the spatial distribution within the 3D spheroid, in relation to spheroid size, is challenging.

Gross qualitative differences can be detected by visual assessment of micrographs. But accurate and reproducible quantification of statistically significant differences requires automated image analysis, especially in large-scale studies. To extract all spatial information from a 3D sample, it is necessary to apply 3D image analysis, but this is a computationally expensive process. Moreover, image resolution is much poorer along the axial direction, so there is a limited information gain by treating the z axis as equivalent to the others. To circumvent this, many methods apply a hybrid form known as 2.5D analysis [13, 14]. Typically, a 2D projection, which by itself sacrifices all depth information, is combined with a method to extract and store a limited amount of depth information. In order to get meaningful information out of 2.5D image analysis, these methods then need to incorporate assumptions that permit extrapolation back to three dimensions. Orthogonal validation, using independently generated "ground truth" is essential to ensure that the analysis sufficiently represents the original data set.

Accurate extraction of quantitative features requires adequate quality images as a starting point. Acquisition of such 3D image data sets is complicated by a number of issues that are less important in, or absent from a 2D setting. These include spherical and chromatic aberrations (which, for a limited sample thickness, are usually corrected for in objective design), poor axial resolution, photobleaching and phototoxicity during long data acquisitions, and light absorption and scattering resulting in signal attenuation deeper into the sample. In this paper, we will focus on how to address the experimental errors due to light attenuation.

In many applications, the effects of light attenuation can be reduced. Tissue samples can be chemically cleared before imaging by using mounting media such as SeeDB [15], Clarity [16], ClearT [17], or Scale [18]. As the effects of light scattering are reduced at longer wavelengths, image quality is greatly improved by multiphoton microscopy, which uses infra-red excitation light that penetrates deeper into tissue [19]. Light-sheet fluorescence microscopy reduces the impact of light attenuation by illuminating and measuring a sample from multiple angles [20, 21]. However, most of these technologies are incompatible with medium and high throughput experiments on hundreds or thousands of samples. These currently need to be performed using standard high-content imagers and microtiter plates. This limits the options to spinning disk confocal microscopy, the use of far-red dyes, and the application of software techniques to correct for light attenuation after acquisition [22, 23].

Diverse tools for the analysis of fluorescent images of 3D spheroid cultures, such as the quantification of proliferation markers used here, are available. General purpose image analysis tools include both commercially available packages, such as, Imaris (Bitplane AG, Switzerland, http://bitplane.com) and Volocity (PerkinElmer, Inc., USA), and freely available open source software tools such as FIJI (ImageJ) [24] and ICY [25]. Moreover, in the context of multicellular spheroids and fluorescent spot detection, multiple tools compatible with high throughput analysis, and dedicated to specific tasks, exist: Amida (spheroid morphology) [26], AnaSP (spheroid volume)[13], MINS (nuclear segmentation) [27], CellSegm (general segmentation)[28], smart 3D-FISH (spot detection) [29], and goIFISH (spot detection) [30]. However, there is no analysis tool to date that addresses spheroid signal attenuation, and can be used in a high throughput fashion. In this paper, a 2.5D image analysis method for 3D spheroid cultures, suitable for Z-stack data acquired by confocal high-content imagers, is presented. Our method segments the entire 3D spheroid and detects the single labeled cells within. These cells are stained with a specific marker, for example a marker for cell proliferation such as EdU. It is conveniently applicable to medium throughput experiments to assess cancer cell growth under varying culture and treatment conditions. The performance of the proposed approach is compared with a simple 2D MIP analysis method, and a full 3D analysis executed using Bitplane Imaris.

## Results and Discussion

The proposed analysis method consists of two major parts: the segmentation of the cancer spheroids and the identification of positive cells, based on fluorescent labeling present in all cancer cells and a specific marker, respectively. Both are explained in detail in the subsequent sections. In our example data set, the cancer cells are labeled with RFP and the specific marker is EdU, a marker for proliferating cells, as detailed in the Materials & Methods section. The parameters and output features of the method are given in Tables 1 and 2.

**Table 1:** Input parameters of the algorithm.

**Algorithm step**	**Parameter**	**Default value**
General	**Average cell diameter:** The expectation value of the cell diameter	12 μm
2D segmentation	**max**_**z-range**_: Threshold on the range-filtered height view	Calculated from the histogram of the range-filtered height view.
	**r**_**range**_: Neighborhood radius for the range filter	3 μm (one-fourth of the cell diameter)
	**min**_**MIP**_: threshold on the mean intensity value of the spheroids	Mean of the background intensity of the MIP
3D ellipsoid approximation	**min**_**radius**_: The z-dimension of spheroids with radii below this threshold is put to the mean radius in the plane instead of determined from the 3D signal.	24 μm
Attenuation	**Intensity attenuation percentage** (threshold): The minimal percentage of the signal that has to be retained to be analyzable	50%
Spot detection	**Maximal spot radius**: Maximal expected spot radius, larger spots are split	8 μm (average nucleus diameter)
	**Average spot radius**: Scale for the spot detection	Optimal scale calculated from the LoG of the MIP

**Table 2:** Output features of the algorithm.

**Feature**	**Description**
Spheroid center	The coordinates of the 3D ellipsoid center of the spheroid
Ellipsoid axes	The axes and their dimensions of the fitted ellipsoid
Spheroid mean intensity	The mean intensity of the 2D spheroid mask
# spots per spheroid	The number of positive cells detected in a spheroid
Spot center	The coordinates of the center of a detected positive cell
Spot intensity	The intensity in the original image of the spot
Circularity	A Podzceck-shape factor determining the resemblance of the spheroid contour to a circle
Roundness class of the spheroid	Elliptical or non-elliptical
Attenuation class of the spheroid	Full, half, or not analyzable spheroid
Spheroid area	Area of the 2D spheroid mask
Spheroid volume	Volume of the 3D ellipsoid

### Segmentation of multi-cellular spheroids

The first step is a 2D segmentation of the spheroids on maximum intensity projections (MIPs). Subsequently, ellipses are fitted to the segmented 2D spheroid-masks. Finally, these ellipses are extrapolated to 3D ellipsoids. The procedure assumes that the 3D image stacks are corrected for uneven background illumination, to eliminate any systematic bias in the height view of the background pixels, and the distance between two subsequent slices (often referred to as z-step) should be less than the average diameter of a cell.

#### 2D segmentation

**Fig 1:**
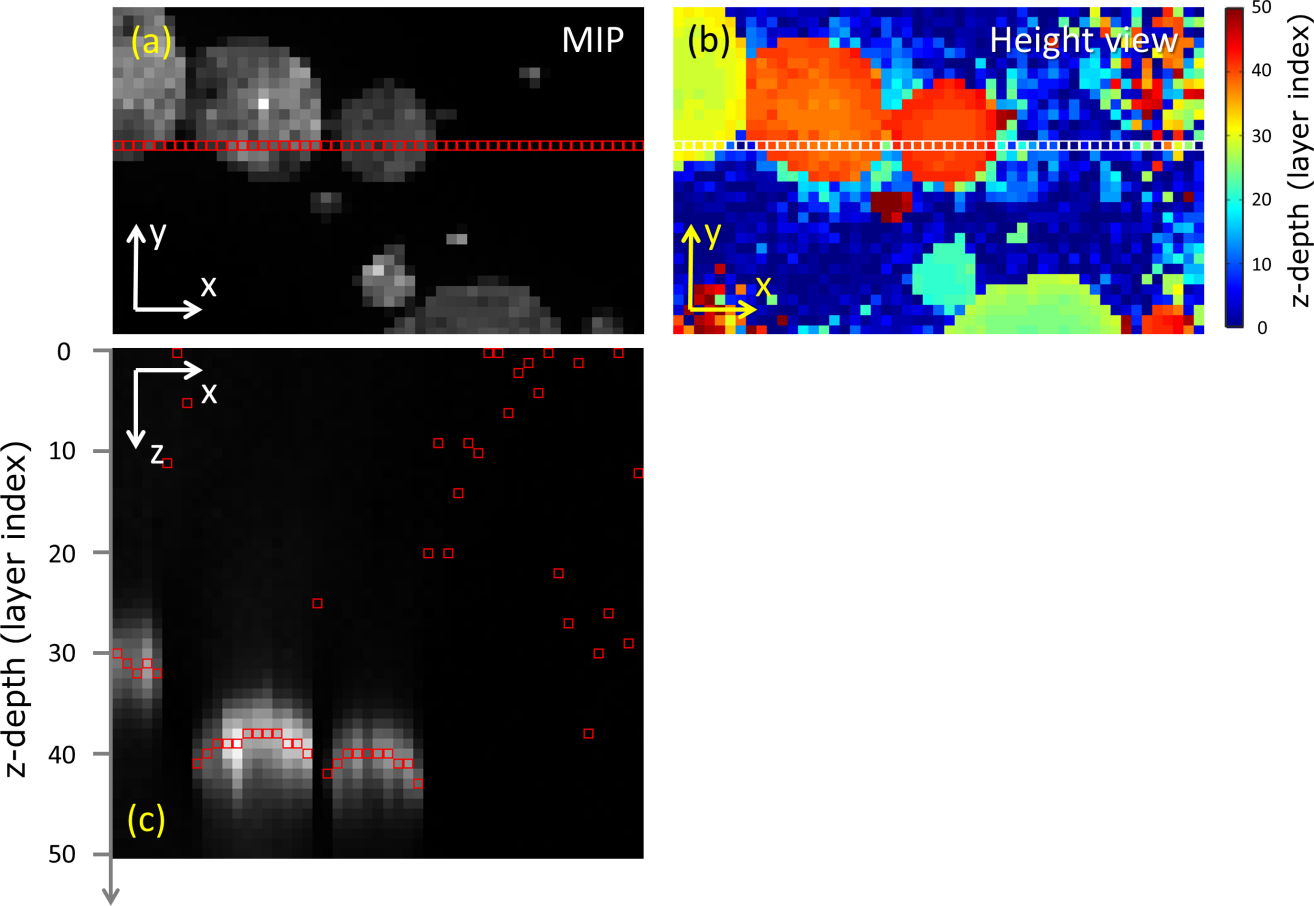
MIP and corresponding height view. The Maximum Intensity Projection (MIP) and the height view of an image are shown (a-b) and for a single xz-plane from the image (c) it is indicated (by red/white squares) where the pixels originated from, that is, which z-coordinates resulted in the highest intensity. The corresponding z-values get mapped to the height view, while the pixel intensities are mapped to the MIP. When two spheroids are overlapping in the lateral direction, the MIP will show the spheroid with the highest intensity.

**Fig 2:**
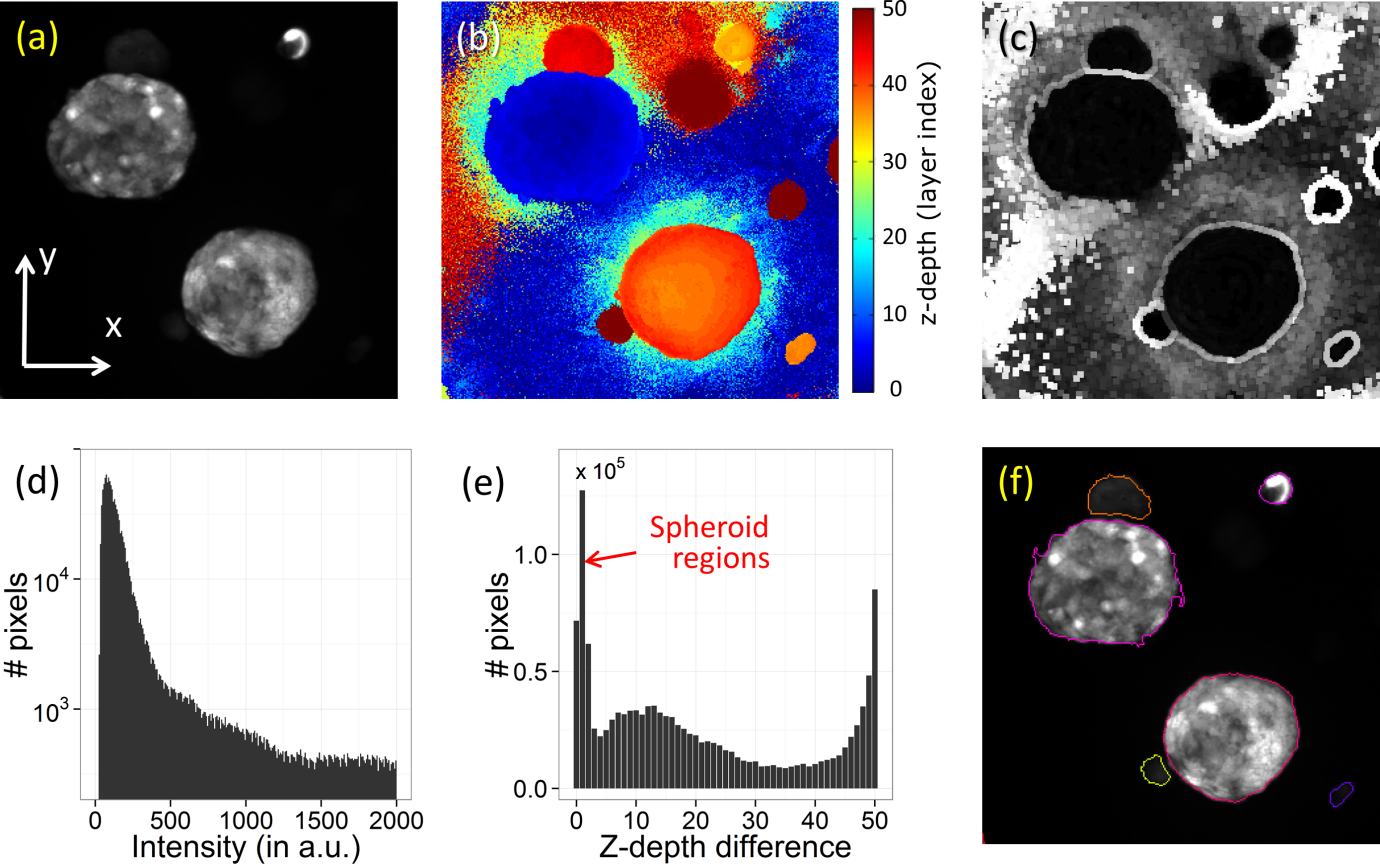
Segmentation of the spheroids in 2D. (a): Part of the maximum intensity projection image of the RFP channel of a 3D image stack, (b): corresponding height view, (c): After applying a range filter on the height view, giving the local variance in the z-depth, where bright (dark) pixels indicate background (spheroid) regions, (d): histogram of (a), showing that the foreground cannot distinguished clearly, (e): histogram of (c), showing that separation between foreground and background is possible. (f) after segmentation.

Fig 1 illustrates the concepts of the MIP and corresponding height view. At each (x,y) coordinate, we obtain the highest intensity value and z-coordinate of the corresponding voxel. For the basic segmentation of the spheroids, which is illustrated in Fig 2, we first select locally homogeneous regions from the height view (shown in 2(b)) [31]. Such regions correspond to object (spheroid) pixels while background pixels tend to be more random in z-coordinate. By applying a range filter, which calculates for every pixel the maximal intensity difference of the neighboring pixels within a radius r_range_, a measure for the local inhomogeneity is obtained, as shown in Fig 2(C). Fig 2(E) shows the histogram of height differences in Fig 2(C), indicating that the large number of pixels at the lower values belong to the spheroid regions. Applying a threshold max_z-range_ on the image in Fig 2(C), a value determined by the extent of the first peak in the histogram, gives rise to separation of objects from background. Finally, the resulting foreground objects are identified using connected-component labeling. Another threshold min_MIP_, determined by the mean of the background MIP intensity, is applied on the mean intensity of each object in the MIP to ensure that the detected objects have an expected minimal fluorescence intensity, characteristic for the spheroids. Applying such a threshold prevents the detection of spurious objects.

#### Fitting of ellipses to 2D spheroid-masks

The fitting of ellipses to the spheroids segmented in 2D is done by minimizing the mean square error of the difference with the segmented 2D mask. During this step, we assume that the elliptical spheroids are verified by measuring the circularity of the 2D segmentation masks of the spheroids. Spheroid-masks with low 2D circularity do not have a 2D ellipse shape, and will not give rise to a satisfying 3D ellipsoid fit. There may be multiple reasons, beyond actual irregularity in spheroid shape, that can contribute to the production of irregular 2D masks. These include spheroids which are not fully visible in the MIP because they are obscured by other brighter spheroids, and spheroids that were incorrectly segmented during the 2D segmentation step, for example, by merging two spheroids. Since these spheroids would give rise to an incorrect 3D segmentation mask, they are filtered out based on their 2D circularity.

#### 3D ellipsoid approximation

**Fig 3:**
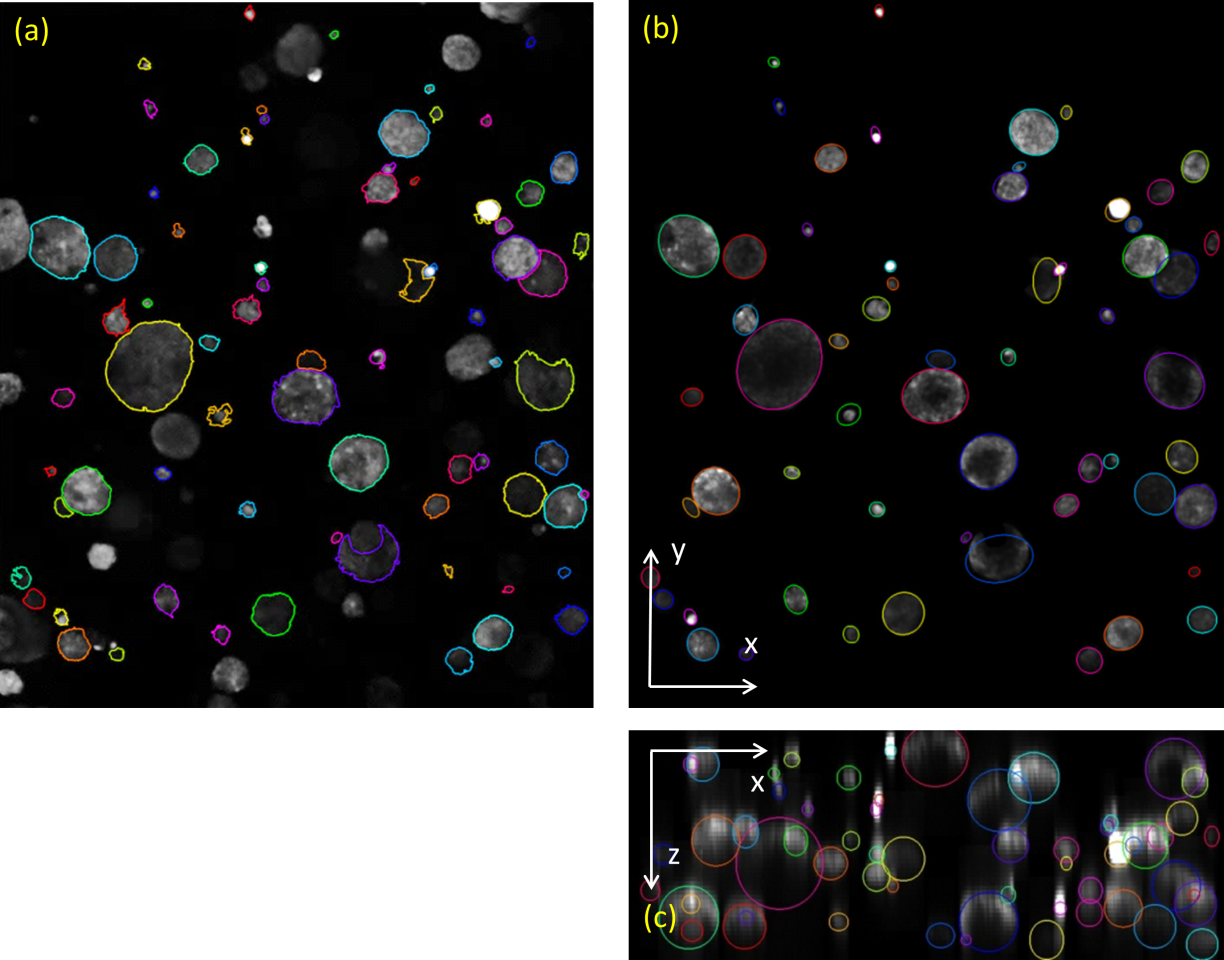
Fitting of 3D ellipsoids. (a) The segmented spheroids in 2D, overlaid on the MIP. (b-c) Ellipsoids fitted to the spheroid mask in (a). The center slice projections of the spheroids from (b) the top (the xy-plane) and (c) the side (the xz-plane), are shown overlaid with the fitted ellipsoids.

Next, a 3D ellipsoid is fit to each of the 2D ellipses. In employing this procedure, we assume that the spheroid has an ellipsoidal shape, which is either nearly spherical, or satisfies the less stringent condition that two of the ellipsoid axes are oriented in the xy-plane. The former case is applicable to spheroids cultivated in a homogenous matrix, and the latter case can occur for spheroid cultures seeded in a layer sandwiched between two matrices. As we assume that the 3rd ellipsoid axis is along the z-axis, the ellipsoid center position and length of the 3rd axis has to be estimated. The (x,y)-coordinates of the center of the ellipsoid in 3D are assumed to be the (x,y)-coordinates of the center of the 2D ellipse, the z-coordinate of the center and the 3^rd^ ellipsoid axis length are derived from the intensity profile along the vertical axis. Fig 3 shows the resulting fitted ellipsoids for an image stack of a 3D spheroid culture. The spheroid volume can now also be estimated.

#### Determination of the analyzable ellipsoid region

**Fig 4:**
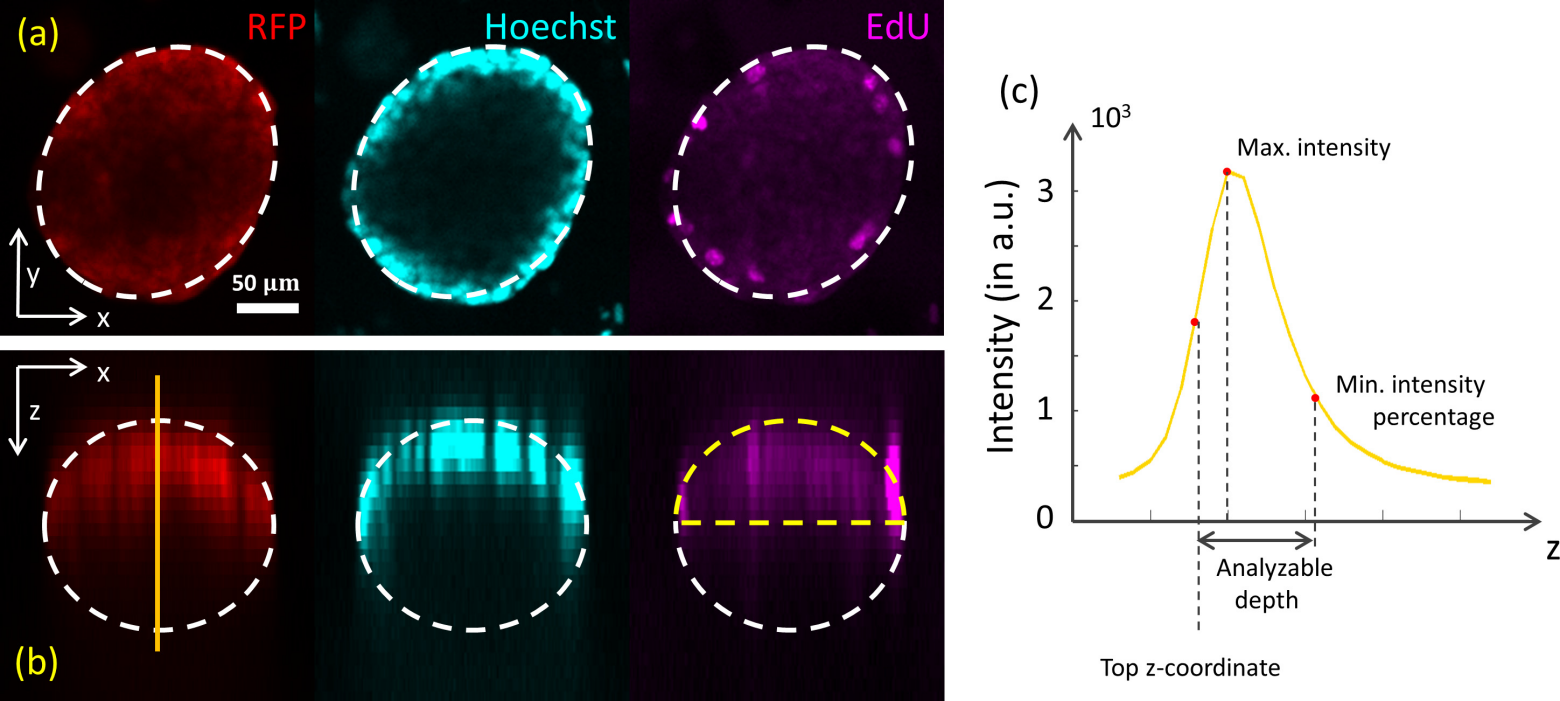
Light attenuation in multi-cellular spheroids. (a) Top view from the xy-plane of a spheroid with an ellipsoid fitted, where the RFP (561 nm), Hoechst (405 nm), and EdU (640 nm) channels are shown. (b) The same spheroid, in a side view (xz-plane). No signal is detected from the lower part of the spheroid (assuming that the spheroid is of an ellipsoidal shape). (c) Parameters of the spheroid derived from the vertical profile curve of the RFP signal through the ellipsoid center: top z-coordinate, maximum intensity, analyzable depth (corresponding to the user-defined minimum intensity percentage).

There is a decreasing intensity gradient from the surface towards the center of a spheroid. Therefore, cells can be incorrectly identified. This issue is illustrated in Fig 4(A), where xy- and xz-slices through the center of a spheroid show the typical intensity distribution due to signal attenuation. Note that the signal of the EdU channel is also reduced in the lower parts of the spheroid, which renders the detection of EdU positive cells impossible in that region. Notice also that signal attenuation due to deep tissue imaging is not the only reason for signal detoriation. When using for example point scanning microscopes, other factors, such as photobleaching, can also become significant.

As all cells in the spheroids carry a stable fluorescent marker (thus assumed to be constant within a single spheroid), their vertical intensity profile through the center of the spheroid can be used as a measure for the signal attenuation. Indeed, a gradual reduction of the stable marker signal (here corresponding to the RFP signal) with increasing depth in the spheroid tissue is observed in Fig 4(C). In order to measure the maximum depth in the spheroid up to which positive cells can be accurately detected, we assume the attenuation of the specific fluorescent marker to be comparable to that of the stable marker. When the wavelengths of both channels do not differ significantly, this is a reasonable assumption. Given a user-defined value for the minimal percentage of intensity that has to be retained after attenuation of the signal, we can determine the depth of the analyzable region of the spheroid (see the Materials & Methods section).

The analyzable depth is subsequently compared to the spheroid vertical diameter. Three distinct cases are identified (as illustrated in S1 Fig):

1. The entire spheroid is visible. Results are not affected by light attenuation.
2. More than half of the spheroid is visible. The upper hemisphere of the ellipsoid will be analyzed and the results extrapolated on the lower half, assuming similar characteristics.
3. Less than half of the spheroid is visible. The spheroid cannot be analyzed because positive cell counts cannot be extrapolated from the spheroid surface to the core, where the availability of oxygen and nutrients is expected to be different [11].

Since only half of a spheroid needs to be visible, our proposed analysis procedure extends the window of spheroid sizes that can be analyzed, allowing diameters to be approximately twice as large. Also, spheroids that are too large for full analysis (i.e., case three) will be identified and may be omitted from sample analysis. In the case that a significant amount of spheroids has to be omitted, the proposed analysis is not appropriate anymore and other types of analysis of the spheroid cell cultures have to be utilized.

#### Validation of the ellipsoid approximation

In order to test the proposed 2D segmentation step, we compared the results of the automated with a manual 2D segmentation of the data, assessing the sensitivity of the spheroid detection and the accuracy of the resulting contours as defined in detail in the Materials & Methods section. The MIPs of 8 image stacks were manually segmented as described in the Materials & Methods section, and we found an average accuracy of 0.911 ± 0.066, and a sensitivity of 0.928, within the 95% confidence interval [0.909, 0.944]. Sensitivity strongly depends on the threshold min_MIP_, and whether a segmented contour is categorized as spheroid or as noise, especially for spheroids that have a mean intensity close to background. In S2(A) and S2(B) Fig we plotted the sensitivity and the ratio of false positives (FP) and their Wilson score interval as function of the intensity threshold min_MIP_. The sensitivity decreases together with the number of false positives with increasing threshold, as such there is a certain trade-off to consider when setting the threshold value. S2(C) and S2(D) Fig illustrate the mean intensity of the spheroid as function of different categories, indicating that spheroid contours classified as false positives (FP) have generally a low intensity. The accuracy of the segmentation contours of the spheroids which are identified in the GT as “well separated spheroids” is significantly high (an exactly matching contour has an accuracy equal to 1) and shows that the non-homogeneity of the height view is a valid measure for the segmentation of this type of cell cultures.

**Fig 5:**
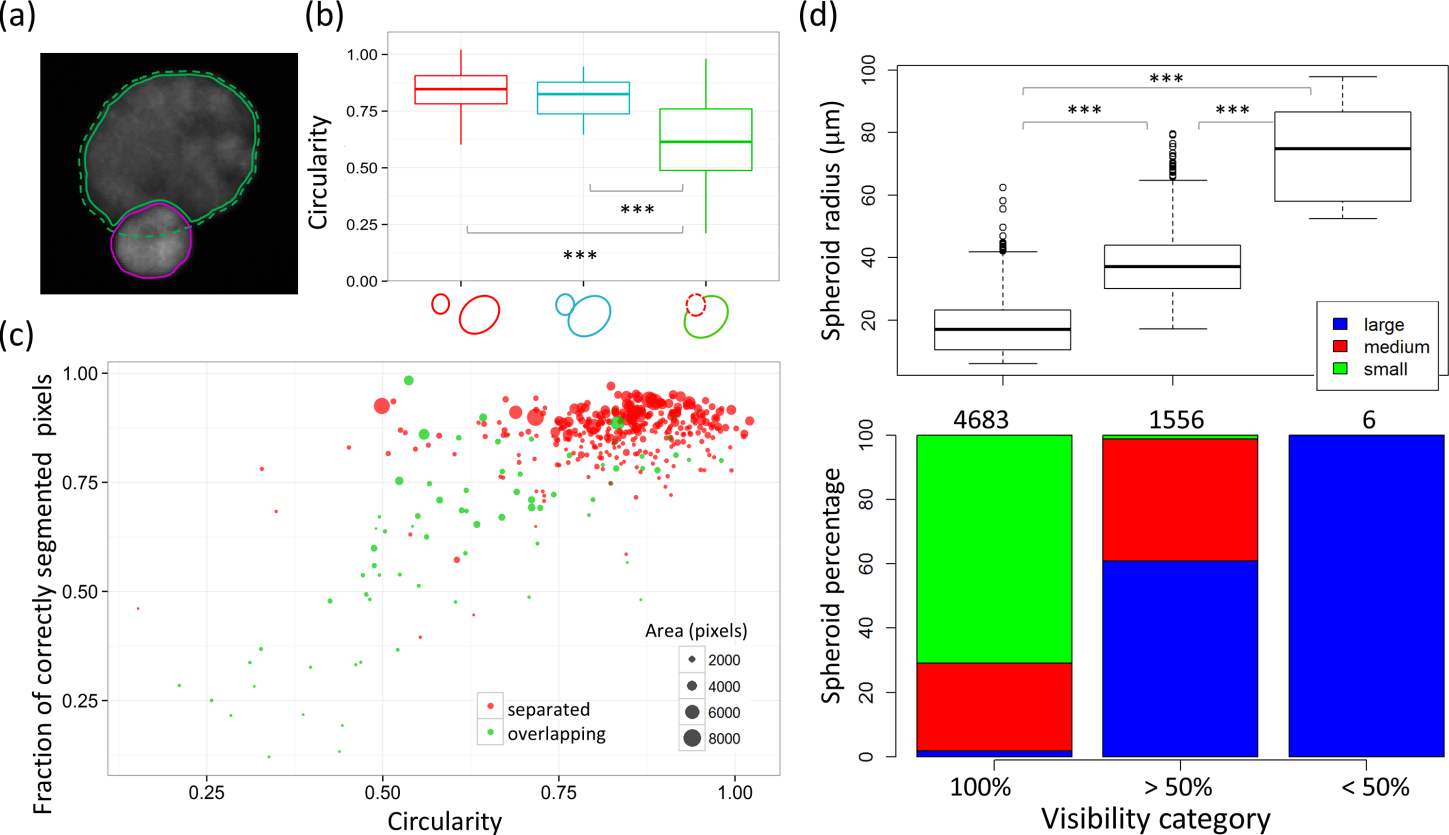
Validation of the ellipsoid segmentation algorithm. (a-c) The classification of spheroids being obscured by others in the GT data is compared with the automated identification of those spheroids based on the circularity of the 2D spheroid masks. (a) illustrates a typical situation where the shape of the 2D spheroid mask is non-elliptical because the spheroid is overlapping with another (brighter) one, the green dashed line shows the GT contour of the spheroid, while the solid contours result from automatic segmentation. (b) shows the spheroid circularity resulting from the automatic segmentation as function of the spheroid class in the GT, where the different classes are given by the cartoons on the x-axis representing well separated (red), adjacent (blue), or overlapping (dark green) spheroids. (c) shows the percentage of correctly segmented pixels by the automatic segmentation as function of the circularity for the class of well separated (red) and overlapping (green) spheroids. (d) shows spheroids of different sizes that are categorized according to their analyzable region: the entirety, more than half, or less than half of the spheroid is analyzable (visibility category). Above the bars the number of spheroids per visibility category is shown. The labels large (blue), medium (red), and small (green) correspond to spheroids with a number of cells larger than 200, between 50 and 200, and smaller than 50, respectively.

Spheroids overlapping in the MIP are unsuitable for analysis (as illustrated in Fig 5(A)). Since the circularity of overlapping spheroids is expected to be lower, identifying spheroids with a small value for circularity should allow for removal of most of these objects. To check whether there indeed is a correlation between overlaps and spheroid roundness, the circularity as determined by the 2D automatic segmentation mask was plotted against the distinct classes of the GT, i.e. well-separated spheroids (red), adjacent spheroids (cyan), and overlapping spheroids (green) in Fig 5(B) where a notable decrease of circularity can be observed in overlapping spheroids (p***).

To verify that a circularity threshold removes overlapping spheroids, the fraction of correctly segmented pixels of the spheroid masks was plotted as a function of their circularity for each spheroid and labeled with the manual classification of the ground truth in Fig 5(C). The correlation between both is highly significant (p***), with an estimated value of r = 0.70 and a confidence interval [0.64, 0.75]. A MANOVA applied on the data indicates that the distributions of the two spheroid classes shown differ significantly (p***). The figure shows further that applying a circularity threshold indeed removes most overlapping spheroids. As an undesired side-effect of this filter, some well separated but non-elliptical structures were removed. These may represent invasive spheroids rather than compact micro-tumors and are excluded from analysis. In S5 Fig the origin of the spheroids which are omitted due to too low circularity is indicated. For the ground truth images, under consideration, less than 20% of spheroids were omitted, primarily due to being obscured during imaging.

Finally, in Fig 5(D), the relation between spheroid size and light attenuation is illustrated, using a sample set containing control and SOC (standard of care) treated cultures, as described in the Materials & Methods section. As in the previous section, the spheroids were categorized into groups of 100%, more than 50%, or less than 50% visible spheroids. While a standard 2D MIP image analysis method would only be able to analyze spheroids in the first 100% visibility category, our approach also allows the quantification of spheroids belonging to the second category (> 50%), which in this case contains approximately 25% of the total number of spheroids, as can be seen from the spheroid count per visibility category.

### Identification of cells positive for a given nuclear marker

Cells positive for a given nuclear marker are detected either via a computationally expensive 3D or a fast 2.5D spot detection algorithm based on the MIP and height view of the corresponding fluorescent channel. It is assumed that resolution is sufficient to resolve the individual nuclei, which are physically separated by the cytoplasm (as visualized in S1 Movie). In both approaches, marker positive spots are found using a Laplacian of Gaussian (LoG) filter, at the scale of the expected size of a nucleus, which reveals the coordinates of the spots in the original image as local minima. The maximal spot radius parameter is set to permit merging of multiple local minima occurring within a nucleus, while maintaining the distinction between separate nuclei. The optimal scale for the expected size of the nuclei is determined beforehand by using the 2D MIP. The total sum intensity of the LoG of the MIP is maximized by optimizing the scale parameter, between one tenth of the maximal spot radius and the value itself. A curve for the total sum intensities as function of the scale parameter is shown in S3 Fig Afterwards, the spots are filtered based on their intensity, with the threshold equal to the median spheroid intensity in the MIP (resulting in a single threshold per image). As an example the results of the approach are shown in panel (b) of S3 Fig where the red circles represent detected EdU positive cells. For the 2.5D spot detection algorithm, the original image stack is exchanged for its 2D MIP and the z-coordinate of a spot is obtained from the height view.

### Application of the approach to a proliferation study

**Fig 6:**
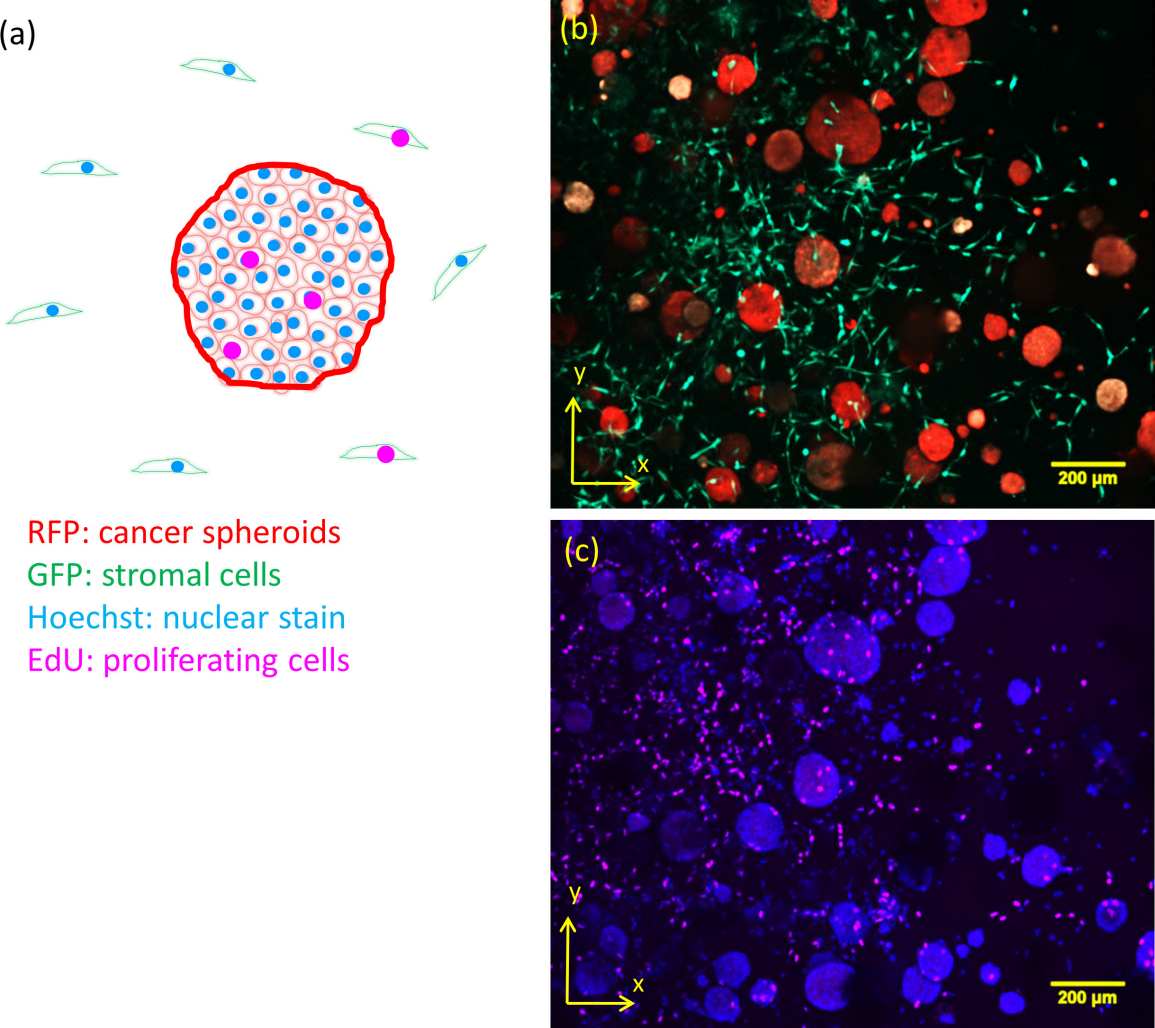
Proliferation analysis example data. (a): Scheme of the labeling of the cells and dyes used. (b-c): Image of a 3D in vitro cancer cell culture labeled / stained with EdU, RFP, GFP and Hoechst. The maximum intensity projection is shown.

**Fig 7:**
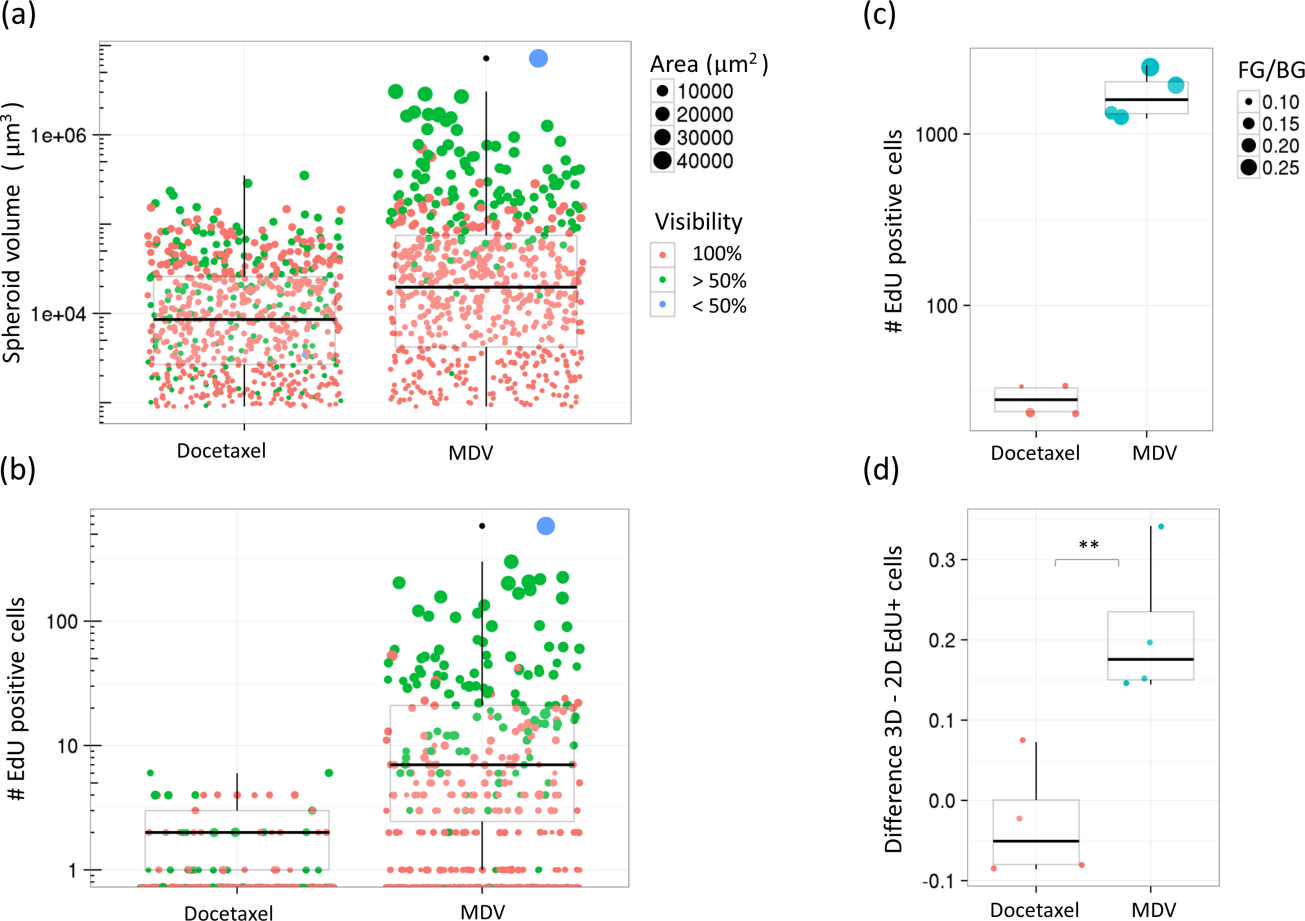
Comparison of the proposed analysis method with a baseline 2D MIP analysis method. Spheroid cell culture proliferation, quantified by EdU positive cells, from a 3D homogeneous spheroid culture treated with a cytotoxic compound (Docetaxel, 1e-8 M) and a cytostatic compound (MDV-3100, 1e-7 M). In (a) the spheroid volume is shown, and in (b) the number of EdU positive cells per spheroid. In (c) the total number of EdU positive cells per image volume is shown, where the size of the dots represent the total foreground / background ratio of the MIP of the image stack. In (d), the EdU positive cell count, based both on the proposed and a 2D MIP analysis method, is compared. The normalized difference in EdU positive cells, weighted with the spheroid volume, is shown.

We applied our proposed algorithm to analyze images of proliferating cells in 3D spheroid cultures, demonstrating its performance on partially attenuated images stacks. These are images of multi-cellular spheroid prostate cancer cell cultures treated for 17 days with a cytotoxic compound (docetaxel) or a cytostatic (MDV-3100) compound. As illustrated in Fig 6, tumor cells were labeled with RFP, stromal cells with GFP, and Hoechst and EdU dyes were applied to identify nuclei and proliferating cells, respectively. The spheroid volume and the number of EdU positive cells per spheroid were analyzed. The volume and number of EdU positive cells per spheroid at the endpoint of the experiment is shown for a culture of PC346-c cancer cells for both compounds in (Fig 7A and 7B). Under docetaxel treatment both volume and number of EdU positive cells per spheroid decrease. The total number of EdU positive cells per image volume (four different fields were imaged in the sample well), shown in Fig 7(C), indicates further that treatment with docetaxel reduces the total number of EdU positive cells in the culture.

#### Comparison with 2D analysis results

An important difference between our approach and a standard 2D MIP analysis (as described in the Materials & Methods section) is that the number of detected EdU positive cells within a large spheroid is corrected for. The significance of this difference is shown in a comparison of the two approaches in the plots in Fig 7(D). As expected, a significant nonzero difference between the two approaches is found in the MDV treated cultures representing a culture with larger spheroids. Our proposed new algorithm corrects for signals lost due to light attenuation, whereas small spheroids basically lead to the same results. As such, our approach is especially useful for spheroid cultures containing a fair amount of larger spheroids in the cultures. In particular this can lead to a significant difference when comparing two samples containing different average spheroid sizes, large vs. small, for example when imaging a culture of growing spheroids at different time points. A bias in the sample of large spheroids from a 2D MIP analysis could then lead to inaccurate conclusions.

#### Comparison with a full 3D analysis

We compared the proposed algorithm with a full 3D analysis executed in a widely used general purpose commercial tool, Bitplane Imaris (Bitplane AG, Switzerland, http://bitplane.com). We compared the detection of proliferating cells and the computation of the volume of matching detected spheroids. Due to differences in segmentation algorithms, the overlap of detected spheroids was 63%. In S4(A) Fig the volumes from both methods are compared. The volume obtained by a full 3D approach is on average larger than in the 2.5D approach for small spheroids, but smaller for larger spheroids. In panel (b) this is illustrated. We hypothesize that due to the intensity-based segmentation in Imaris, very small spheroids tend to appear stretched along the z-direction because of poor z-resolution, increasing the total volume, while larger spheroids tend to be not fully imaged due to signal attenuation. To test this hypothesis we plotted the sphericity of the spheroids as function of their volume in panel (c). The decrease in the fitted curve indicates that the larger spheroids are not correctly segmented. To illustrate the effect on a growth analysis we plot the difference between the number of EdU positive cells obtained by our approach and Imaris. This plot in panel (d) indicates that for larger spheroids (where the visibility category is less than 100%), our approach results in more spots, corresponding to the correlation coefficient (Spearman) which was equal to 0.26.

To demonstrate the computational advantage of taking a 2.5D approach we timed both methods (on a standard workstation with an Intel^®^ Core™ i7-3920XM CPU running at 2.9 GHz and 8 GB RAM). The image stacks used had a size of 1024 x 1256 x 91 pixels (two 16bit channels were used). In Imaris it took on average 7 minutes to perform both the spheroid segmentation and spot detection. Our 2.5D approach executed this in 3 minutes and when the simplified spot detection algorithm (based on the MIP and height view) was used this reduced to 1 minute.

## Materials and Methods

### Multi-cellular spheroid culture image stacks

The cell cultures, used for testing the analysis method, contained LNCaP human prostate cancer cells (ATCC, Rockville, USA) or PC346c cells (a prostate cancer cell line obtained from the Erasmus Medical Centre, via the PREDECT consortium) and CAF-PF179T human cancer associated fibroblasts (a cell line obtained from the Weizmann Institute, via the PREDECT consortium) [32], embedded in Matrigel^®^ (Corning #356231, Lot#3198769, growth factor reduced, phenol red free, concentration: 4 mg/ml). They were suspended in the matrix and seeded (10,000 tumor cells and 1,000 stromal cells in 60 μl/well) in 96 well plates (96 well polystyrene cell culture black microplates with a μClear^®^ bottom; Greiner, #655090), which were pre-coated with matrix (30 μl/well). Treatment of cultures with either a cytotoxic compound (docetaxel), or a cytostatic compound (MDV-3100) started on day 6, when the cultures were in exponential phase, and the experiment was ended at day 23, when untreated cultures had reached stationary phase. Cancer and stromal cells stably expressed tRFP and eGFP, respectively. Prior to imaging, cell cultures were stained with 10 μM EdU for 2h (Click-iT EdU Alexa Fluor 647 HCS; Life Technologies) to detect proliferating cells, and Hoechst 33342 (8.1 μM, 30 min) to label all nuclei and then fixed with 4% formaldehyde.

Microscopic image stacks of multi-cellular spheroid cultures were acquired with a Yokogawa CellVoyager 7000 confocal spinning disk microscope (Wako Automation, San Diego, United States), using a UPLSAPO 10x/0.4 NA objective and a 10 μm z-separation between the slices in the stack. Excitation wavelength–filter pairs used for the tRFP, eGFP, Hoechst, and EdU channel were 561 nm—BP 600/37, 488 nm—BP 522/35, 405 nm—BP 447/45, and 640 nm—BP 676/29, respectively.

### Ground truth images

Ground truth data were generated for 3D homogeneous multi-cellular LNCaP cancer spheroid cultures by manually drawing a 2D mask for each spheroid on the MIP. Moreover, each spheroid was classified to one of the cases: (1) well separated, (2) overlapping with brighter spheroids in the MIP (rendering it non-separable), (3) overlapping with less bright spheroids in the MIP (rendering it well separable), (4) merely touching other spheroids, and (5) touching the border of the 2D projection of the image. The labelling was conducted using the RoiManager plugin in FIJI (ImageJ) [24] resulting in contours for the cases (1) to (5) colored in red, green, magenta, cyan, and blue, respectively. These ground truth data were used to validate the 2D segmentation step of the algorithm by defining sensitivity and accuracy, and are provided in S2 File.

### Implementation of the analysis algorithms

Analysis of the image stacks was conducted using the image analysis toolbox DIPimage (Delft University of Technology, Delft, The Netherlands) for MATLAB^®^ (Release 2014a, The MathWorks Inc., Natick, United States). The algorithms are available as a bundle of MATLAB scripts in S1 File: Image analysis algorithms source code, containing both the code to generate the figures, and the implementation of the method. Running the provided code requires a working MATLAB environment with installed DIPimage toolbox and the following external libraries: the Bio-formats toolbox for MATLAB, JSONlab, and ReadImageJROI. The implementation is also available on GitHub: https://github.com/mbarbie1/ellipsoids-analysis-paper.git.

### Statistical analysis

Statistical analysis was executed in R (https://www.r-project.org) using the RStudio IDE (https://www.rstudio.com). The data was tested for normality using the Shapiro-Wilks test, significances were obtained using Welch’s T-Test and the non-parametric Mann-Whitney-U-Test for non-normal distributions. To compare multivariate distributions, e.g. data for the spheroid size and number of proliferating cells, a MANOVA was applied. In all such cases only two groups were compared, thereby the F-statistic approximation is reduced to the Hotelling's T-square statistic. Significances were depicted as *: p < 0.05, **: p < 0.01, and ***: p < 0.001.

### Derivation of the ellipsoid 3D fit parameters

The z-coordinate of the center and the length of the vertical axis are derived from the pixels of the original 3D image. The z-coordinate of the center of the ellipsoid is defined as the median value of the z-coordinates of the outer rim of the 2D segmentation mask. To derive the length of the vertical ellipsoid semi-axis, we consider the neighborhood which contains the 8 nearest neighbor pixels of the ellipse center (*x*_*c*_, *y*_*c*_) in the xy-plane, and a sufficient range (2 times the largest diameter D of the ellipse) in the z-direction:

$$\left(x,y,z)\in \{x,y,z:\;(x,y)\in [{(x}_{c},y_{c})-(1,1),\;{(x}_{c},y_{c})+(1,1)]\;\&\;z\in [z_{c}-D,\;z_{c}+D]\right\}$$

Averaging over this neighborhood yields a profile I(z) through the centroid of the 2D spheroid segmentation mask (see Fig 4(C)). The z-coordinate of the top of the spheroid (which suffers the least from light attenuation) is defined from the profile curve as the z-coordinate above the spheroid center where the curvature becomes zero (the inflection point). When the 2D mask of the spheroid under consideration has an area below a specific threshold min_radius_, the above approach is not used; instead the length of the vertical axis is approximated by the average length of the ellipse axes in the plane.

### Characterization of the signal attenuation in the spheroids

The depth up to which the spheroid is analyzable is limited by the signal attenuation in the spheroid tissue. We calculate the maximal depth (z-coordinate) in the spheroid for which a user-defined percentage of the intensity is still retained after signal attenuation. To obtain this depth we assume that: (1) the fluorescent stain of the tumor cells is constant within each spheroid, (2) the scattering and absorption coefficients determining the attenuation of the signal are constant inside spheroid tissue and zero outside, and (3) intensity values of the first slice of the spheroid are free of attenuation.

The signal attenuation is expected to be largest in the (x,y)-center of the ellipsoid for each z-coordinate. Therefore, the vertical intensity profile through the center of the ellipsoid is used to characterize the attenuation of the signal. The intensity profiles *I*(*z*) are determined as described in the previous section (see Fig 4(C)). They typically have a steep inclination towards a single maximum *I*_max_ = max_*z*_ *I*(*z*) near the top of the spheroid, followed by an intensity decrease. We consider *I*_max_ the intensity without attenuation, and the percentage retained after attenuation is defined as *P*_att_(*z*) = 100 *I*(*z*) / *I*_max_. The user-defined threshold on the minimal percentage retained *P*_min_ leads to a range [*z*_max_, *z*_analyzable_] for which *P*_att_(*z*) > *P*_min_ is valid. The analyzable depth of the spheroid is defined as the distance between the first z-coordinate of the spheroid and *z*_analyzable_.

### Other analysis methods used for comparison

For comparative purposes, a 2D MIP analysis as well as a full 3D analysis, were utilized. Both approaches are detailed below.

#### Baseline 2D MIP analysis method

For the 2D analysis of proliferation in the test data, the following image analysis approach was applied: Similarly as in the proposed algorithm a 2D segmentation mask of the MIP of the RFP channel was obtained, thereafter EdU positive cells were identified by a 2D spot detection algorithm performed on the EdU channel. But unlike in the proposed analysis, all EdU positive cells within the 2D regions of the spheroid masks are considered to belong to the corresponding spheroids, and no correction for signal attenuation was attempted.

#### Imaris based full 3D analysis workflow

Analysis of the images was conducted using the cell extraction functionality from ImarisCell, a component of Bitplane Imaris (v8.1.2, Bitplane AG, Switzerland, http://bitplane.com), where “spheroids and proliferating cells” translate to “cells and cell vesicles”. Spheroids were segmented using the surface extraction tool and morphological features of the spheroid surfaces were extracted. Proliferating cells were detected using the spot detection tool. Subsequently, the detected surfaces and spots were imported in the cell analysis module to determine the spheroid borders and proliferating cells. Finally, features relating to the location of the spheroids and number of proliferating cells were exported. As a final remark we mention that although an attenuation correction method for homogeneous signal attenuation exists (through the Imaris module ImarisXT), our spheroid cultures are largely inhomogeneous and this method cannot be applied.

### Sensitivity and accuracy of the 2D segmentation mask

To quantify the deviation of an automated segmented mask from the ground truth image, we compare the 2D segmented image regions *R*_*GT*_ = {*M*_*j*_} of the ground truth image with the regions *R*_*A*_ = {*m*_*i*_}, found by automatic segmentation. For each region *M*_*j*_ we search a corresponding region *m*_*i*_ that overlaps with *M*_*j*_ more than 50%. Regions *M*_*i*_ for which no corresponding region is found are considered as false negatives (FN), while regions *m*_*i*_ not belonging to a ground truth region *M*_*j*_ are false positives (FP). The sensitivity is given by the ratio: TP/(TP + FN), where TP stands for true positives. The error on the sensitivity is given by the confidence interval as defined by Wilson, the so-called Wilson score interval [33].

For true positives (segmented regions *m*_*i*_ which do belong to a ground truth region according to the above criterion) the segmentation accuracy can be quantified by assigning a penalty determined by how close the contours of the two regions are. This is done as follows: the distance of the pixels of regions *m*_*i*_\*M*_*j*_ (including all pixels from *m*_*i*_ which are not belonging to *M*_*j*_) and *M*_*j*_\*m*_*i*_, from the contour of region *M*_*j*_ is calculated, using the distance image (in μm) of the contour mask of *M*_*j*_, which weights the error contribution of misclassified pixels. A sigmoid function *f*(*x*) = 1 / {1 + exp[−*k* (*x* − *x*_0_)]} with *x*_0_ = 1.5 μm and *k* = 2 μm^-1^ is applied to this distance, so that very small errors in the spheroid outline contribute little to the error estimate, but large errors have a constant weight. Then the pixels are summed over and normalized by dividing by the area of the ground truth region. The resulting penalty *e*_*i*_ is close to zero for nearly overlapping spheroid masks, and the accuracy of spheroid mask *m*_*i*_ can be defined as 1 − *e*_*i*_.

## Supporting Information

S1 Fig Relationship between the visibility categories and the spheroid size. From left to right spheroids of decreasing sizes are shown, as representatives of the different visibility categories: Less than half of the spheroid, more than half, or the entire spheroid is measurable. xy- and xz-slices through the middle of the spheroids are shown and overlaid with white dashed ellipsoid contours (which were fitted manually). In the xz-planes a manually drawn thin dashed contour shows the approximate boundary of the analyzable region of the spheroids. As spheroid size decreases, the Point Spread Function (PSF) of the confocal microscope leads to distortion of the spherical shape.
(TIF)

S2 Fig Segmentation sensitivity dependency on the intensity threshold min_MIP._ (a-b) The dependency of the sensitivity, the FP/P ratio, and their 95% confidence interval (Wilson) on the threshold set on the mean intensity of the segmented spheroids (min_MIP_). (c) Shows the mean intensity of each spheroid as function of their segmentation category: true positives (red), false positives (green), spheroids missed in the segmentation (blue), segmentation masks merging multiple spheroids in the GT (magenta), and spheroids which are detected as multiple spheroid masks (brown). (d) Represents the number of spheroids for each of the categories.
(TIF)

S3 Fig Spot detection in scale space. The spot detection steps are shown using the example image stack in file Data_3, which is available from Dryad (doi:10.5061/dryad.0m9n7). (a) shows the total sum of pixel intensity of the LoG-filtered MIP image as function of the Gaussian scale parameter σ (smoothing parameter). The σ which maximizes the total sum of pixel intensity (of the negative LoG) represents the optimal scale σ for the spot detection. (b) shows a visual comparison of the results from the spot detection originating from the 3D LoG approach used in our approach (red circles), and, the 3D spot detection calculated using Bitplane Imaris (blue circles).
(TIF)

S4 Fig Comparing the proposed analysis method with a full 3D (using Bitplane Imaris) analysis approach. (a) the obtained spheroid volume for both the full 3D method (blue dots) and our approach (red dots) is shown for corresponding spheroids. (b) the ratio of the volume obtained from Imaris over the one obtained from our approach, together with a linear fit of the data. In panel (c) the sphericity, of the spheroid surfaces obtained in Imaris, is plotted as function of the volume. Here a non-linear fit is obtained. (d) the difference of the number of proliferating cells obtained by our approach with the ones obtained in Imaris are plotted, where the colors correspond to the visibility category: 100% (red dots) and > 50% (green dots). There were no spheroids with a visibility < 50% for these samples.
(TIF)

S5 Fig Spheroids omitted during ellipsoid approximation due to shape irregularity. The percentages of spheroids that are omitted due to low circularity are categorized in spheroids with a non-spherical shape, obscured spheroids or incorrect segmented spheroids. As dataset the ground truth validation images from the file Data_4, which is available from Dryad (doi:10.5061/dryad.0m9n7), are used.
(PNG)

S1 File Algorithm implementation. This is a zip-file containing all the source code files. The code is written in MATLAB using the DIPimage toolbox. The source code is also available on GitHub: https://github.com/mbarbie1/ellipsoids-analysis-paper.git.
(ZIP)

S2 File 2D ground truth. This is a zip-file containing manually segmented 2D ground truth labeled masks (the corresponding 3D image stacks can be found in the file Data_4 which is available from Dryad (doi:10.5061/dryad.0m9n7)). The cancer (LNCaP) spheroids are labeled in five distinct classes: (1) well separated, (2) overlapping with brighter spheroids in the MIP (rendering it non-separable), (3) overlapping with less bright spheroids in the MIP (rendering it well separable), (4) merely touching other spheroids, and (5) touching the border of the 2D projection of the image. Opening of the images in FIJI (ImageJ) with the ROI Manager allows visual inspection of the data.
(ZIP)

S1 Movie Spot detection algorithm illustrated on a 3D image stack represented as a movie. This is the 3D image stack from the file Data_6, which is available from Dryad (doi:10.5061/dryad.0m9n7), saved as a movie, in AVI format with JPEG compression. It shows the EdU channel of the 3D image stack in the file Data_3, available from Dryad (doi:10.5061/dryad.0m9n7), with the EdU positive cells annotated by small spheres. This movie can serve as an example result of the spot detection algorithm (3D version).
(AVI)
