# Supplementary figures and images for: Ellipsoid Segmentation Model for Analyzing Light-Attenuated 3D Confocal Image Stacks of Fluorescent Multi-Cellular Spheroids
Source: PLoS One. 2016 Jun 15;11(6):e0156942. doi: 10.1371/journal.pone.0156942 (PMC4909318; doi:10.1371/journal.pone.0156942)

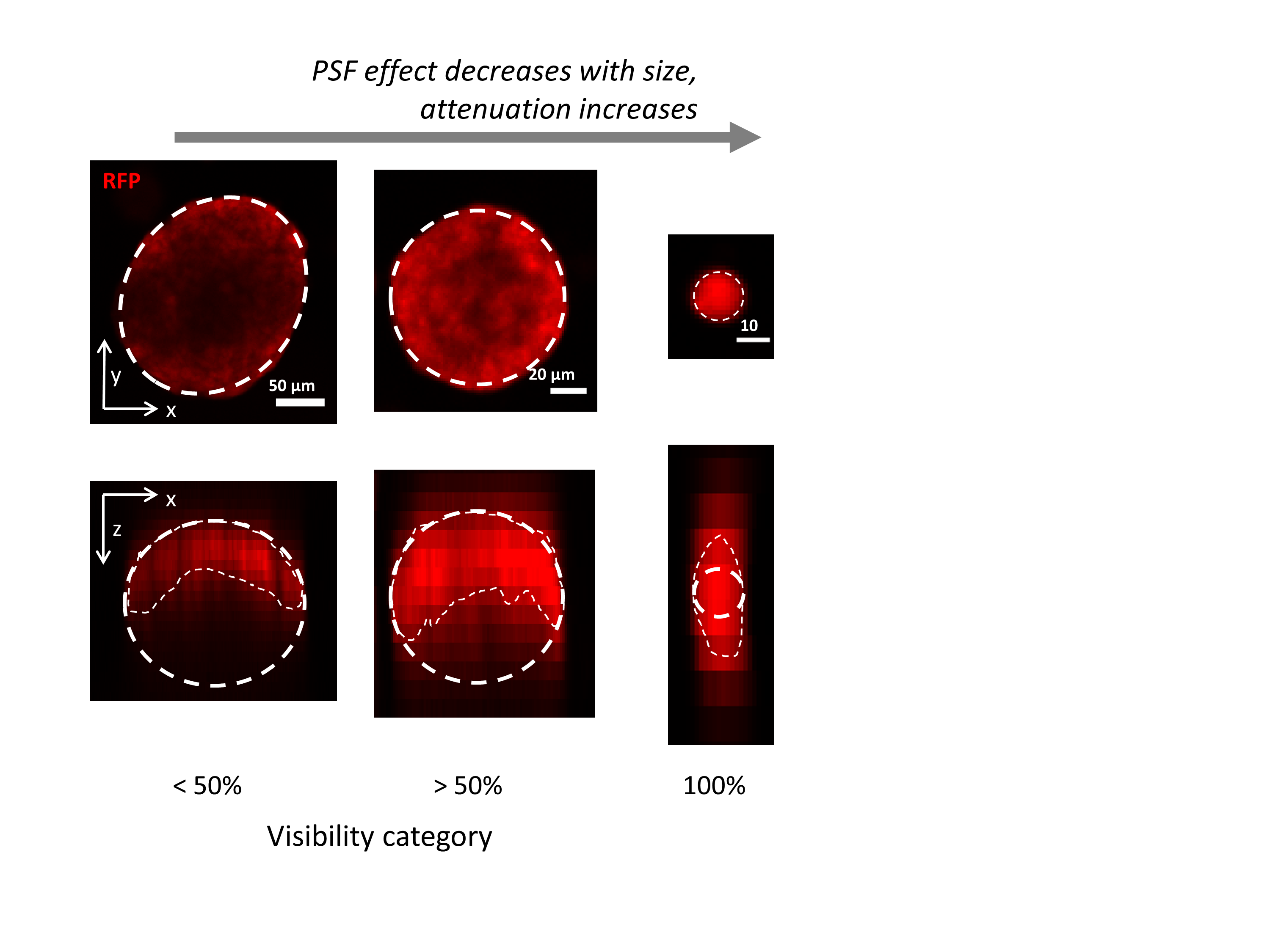

Supplement: S1 Fig — From left to right spheroids of decreasing sizes are shown, as representatives of the different visibility categories: Less than half of the spheroid, more than half, or the entire spheroid is measurable. xy- and xz-slices through the middle of the spheroids are shown and overlaid with white dashed ellipsoid contours (which were fitted manually). In the xz-planes a manually drawn thin dashed contour shows the approximate boundary of the analyzable region of the spheroids. As spheroid size decreases, the Point Spread Function (PSF) of the confocal microscope leads to distortion of the spherical shape. (TIF) [file pone.0156942.s001.tif]

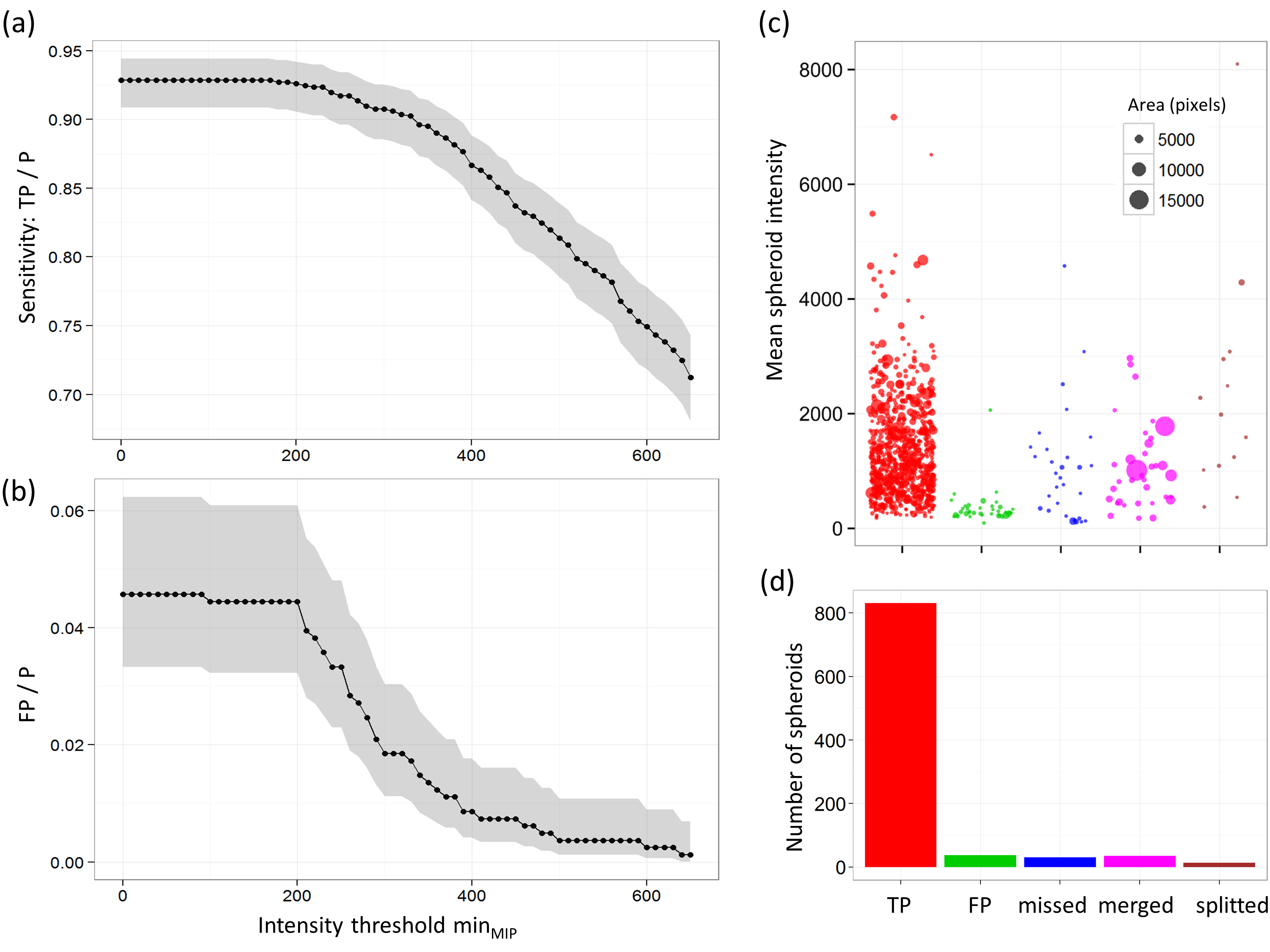

Supplement: S2 Fig — (a-b) The dependency of the sensitivity, the FP/P ratio, and their 95% confidence interval (Wilson) on the threshold set on the mean intensity of the segmented spheroids (minMIP). (c) Shows the mean intensity of each spheroid as function of their segmentation category: true positives (red), false positives (green), spheroids missed in the segmentation (blue), segmentation masks merging multiple spheroids in the GT (magenta), and spheroids which are detected as multiple spheroid masks (brown). (d) Represents the number of spheroids for each of the categories. (TIF) [file pone.0156942.s002.tif]

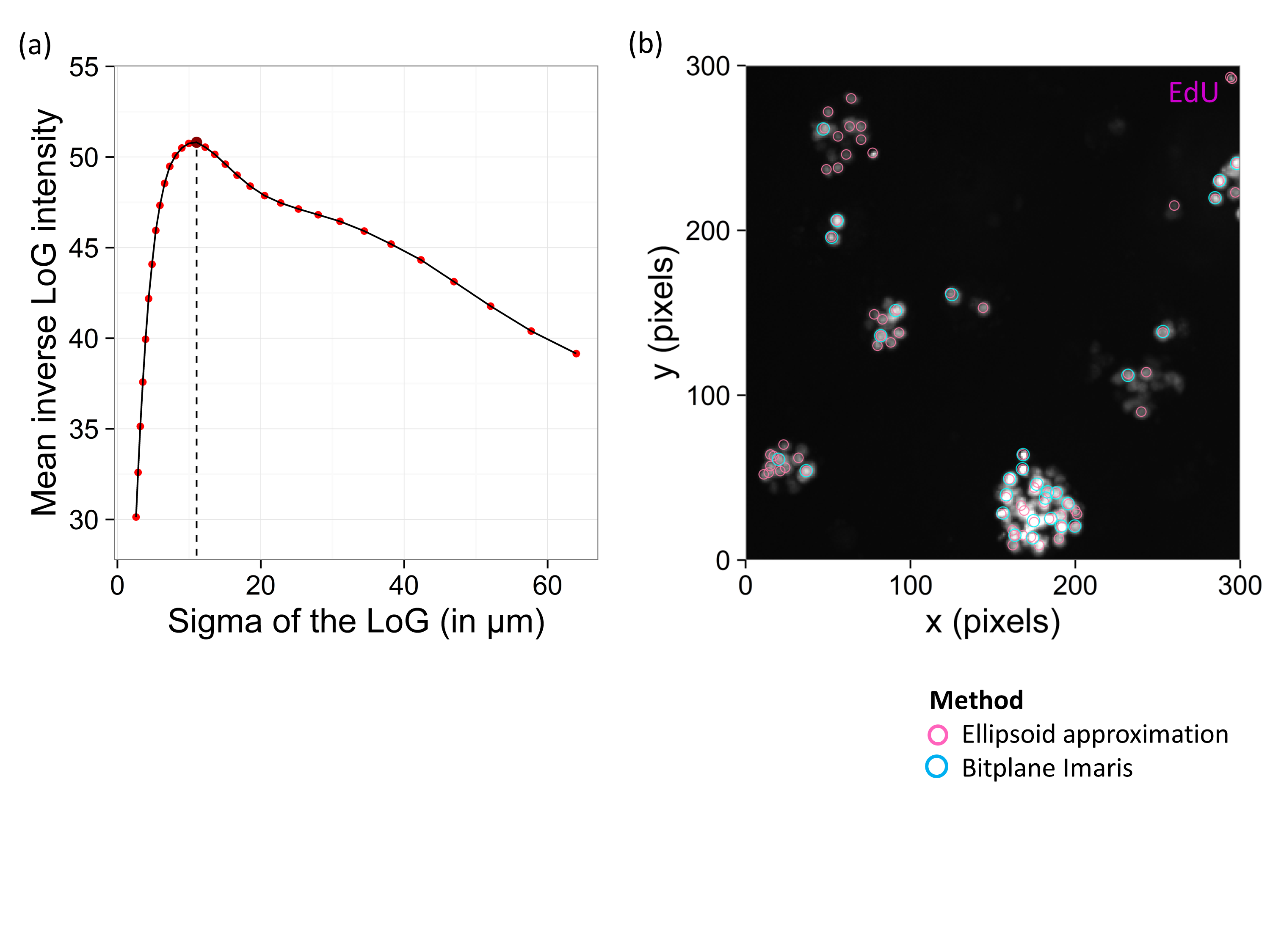

Supplement: S3 Fig — The spot detection steps are shown using the example image stack in file Data_3, which is available from Dryad (doi:10.5061/dryad.0m9n7). (a) shows the total sum of pixel intensity of the LoG-filtered MIP image as function of the Gaussian scale parameter σ (smoothing parameter). The σ which maximizes the total sum of pixel intensity (of the negative LoG) represents the optimal scale σ for the spot detection. (b) shows a visual comparison of the results from the spot detection originating from the 3D LoG approach used in our approach (red circles), and, the 3D spot detection calculated using Bitplane Imaris (blue circles). (TIF) [file pone.0156942.s003.tif]

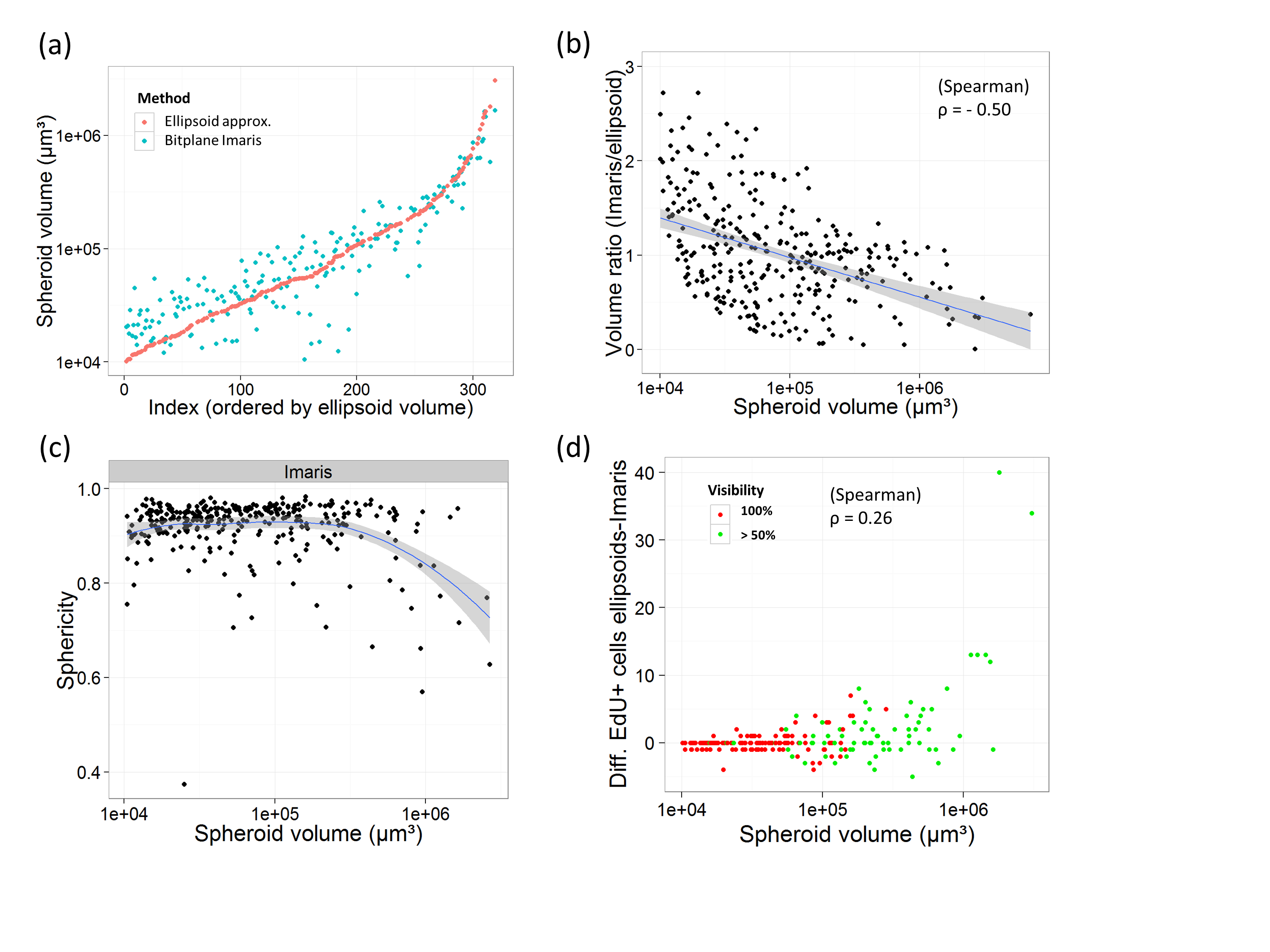

Supplement: S4 Fig — (a) the obtained spheroid volume for both the full 3D method (blue dots) and our approach (red dots) is shown for corresponding spheroids. (b) the ratio of the volume obtained from Imaris over the one obtained from our approach, together with a linear fit of the data. In panel (c) the sphericity, of the spheroid surfaces obtained in Imaris, is plotted as function of the volume. Here a non-linear fit is obtained. (d) the difference of the number of proliferating cells obtained by our approach with the ones obtained in Imaris are plotted, where the colors correspond to the visibility category: 100% (red dots) and > 50% (green dots). There were no spheroids with a visibility < 50% for these samples. (TIF) [file pone.0156942.s004.tif]

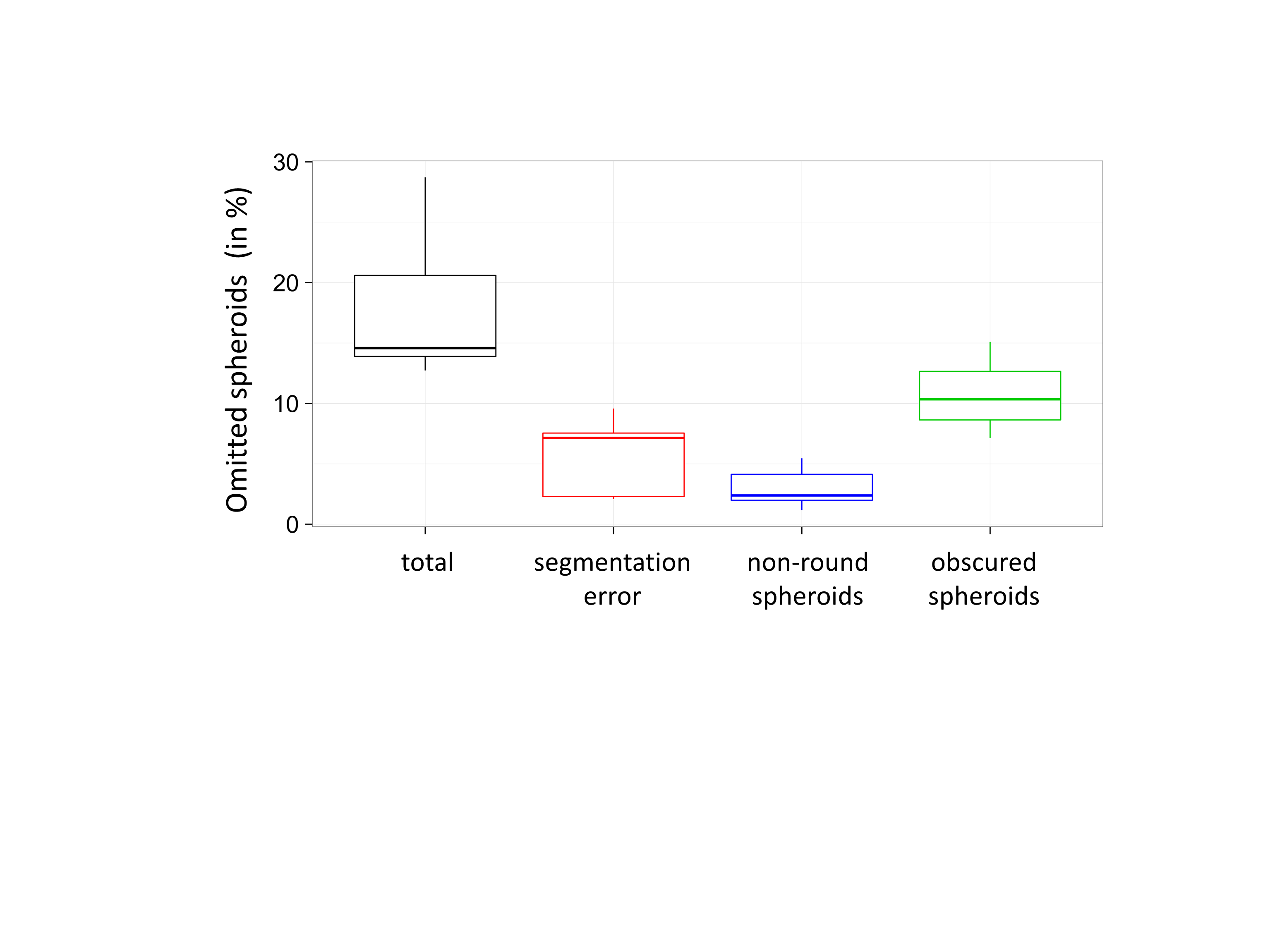

Supplement: S5 Fig — The percentages of spheroids that are omitted due to low circularity are categorized in spheroids with a non-spherical shape, obscured spheroids or incorrect segmented spheroids. As dataset the ground truth validation images from the file Data_4, which is available from Dryad (doi:10.5061/dryad.0m9n7), are used. (PNG) [file pone.0156942.s005.png]

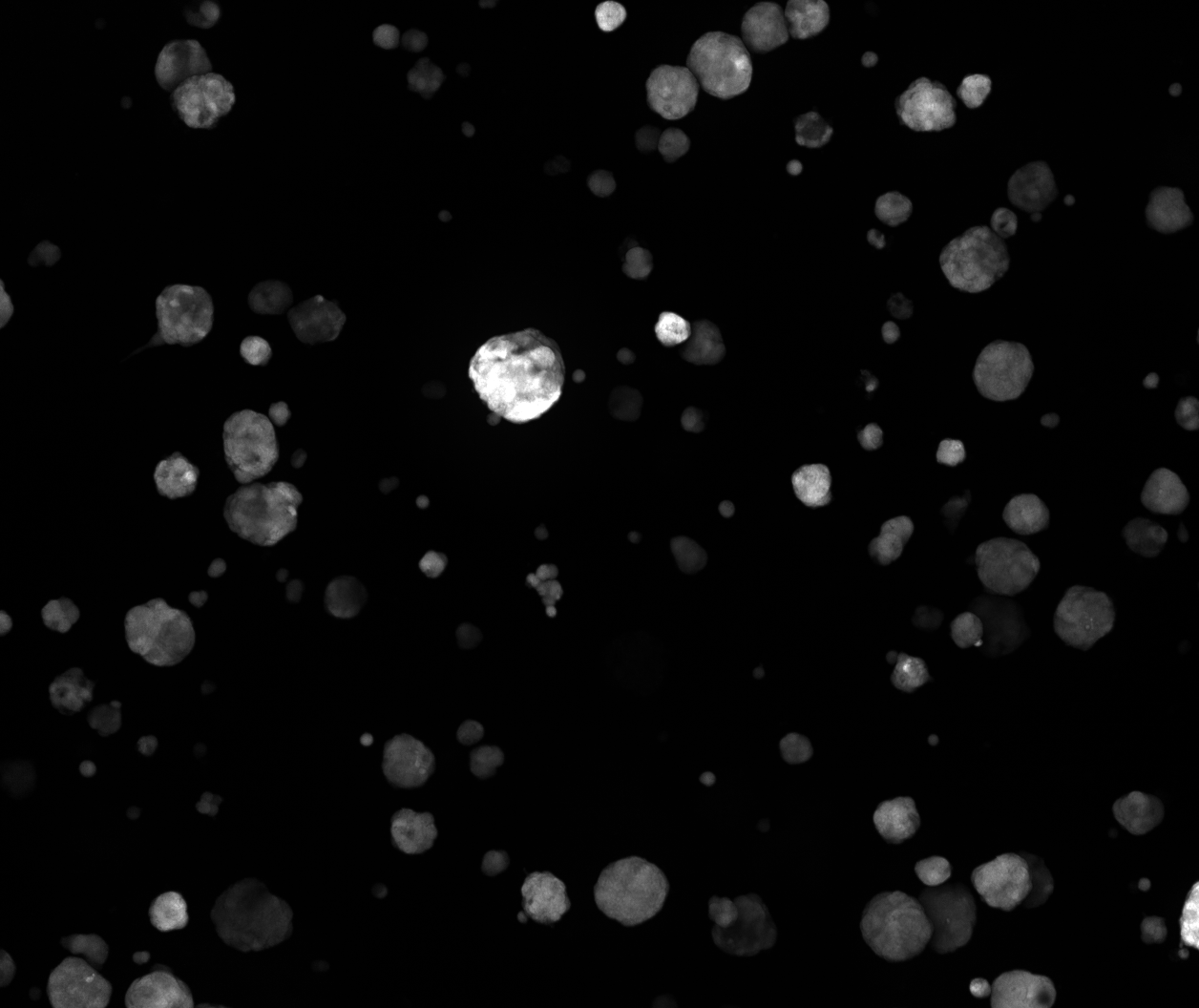

Supplement: S2 File — This is a zip-file containing manually segmented 2D ground truth labeled masks (the corresponding 3D image stacks can be found in the file Data_4 which is available from Dryad (doi:10.5061/dryad.0m9n7)). The cancer (LNCaP) spheroids are labeled in five distinct classes: (1) well separated, (2) overlapping with brighter spheroids in the MIP (rendering it non-separable), (3) overlapping with less bright spheroids in the MIP (rendering it well separable), (4) merely touching other spheroids, and (5) touching the border of the 2D projection of the image. Opening of the images in FIJI (ImageJ) with the ROI Manager allows visual inspection of the data. (ZIP) [file pone.0156942.s007.zip › RoiImage_0333.tif]

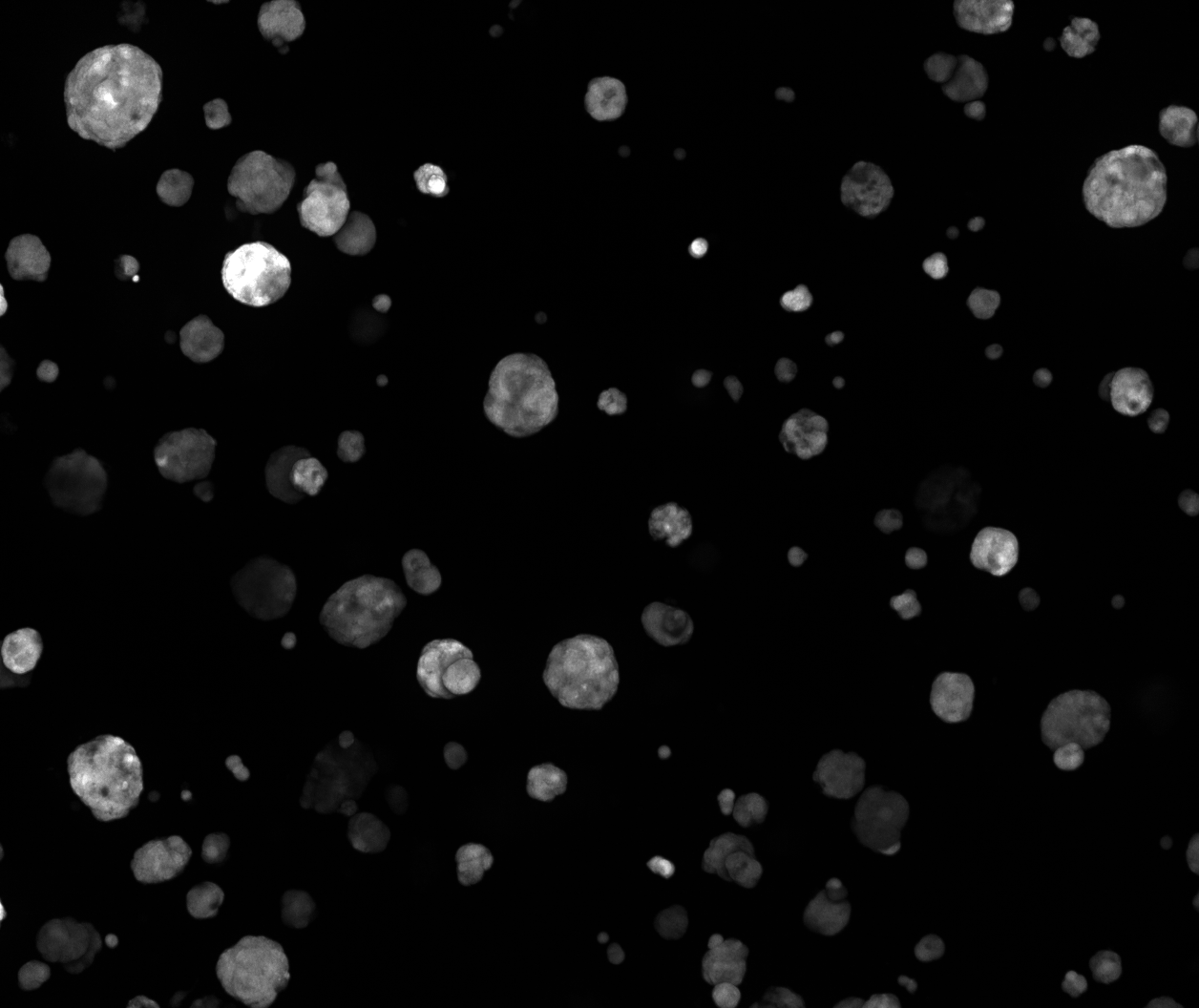

Supplement: S2 File — This is a zip-file containing manually segmented 2D ground truth labeled masks (the corresponding 3D image stacks can be found in the file Data_4 which is available from Dryad (doi:10.5061/dryad.0m9n7)). The cancer (LNCaP) spheroids are labeled in five distinct classes: (1) well separated, (2) overlapping with brighter spheroids in the MIP (rendering it non-separable), (3) overlapping with less bright spheroids in the MIP (rendering it well separable), (4) merely touching other spheroids, and (5) touching the border of the 2D projection of the image. Opening of the images in FIJI (ImageJ) with the ROI Manager allows visual inspection of the data. (ZIP) [file pone.0156942.s007.zip › RoiImage_0334.tif]

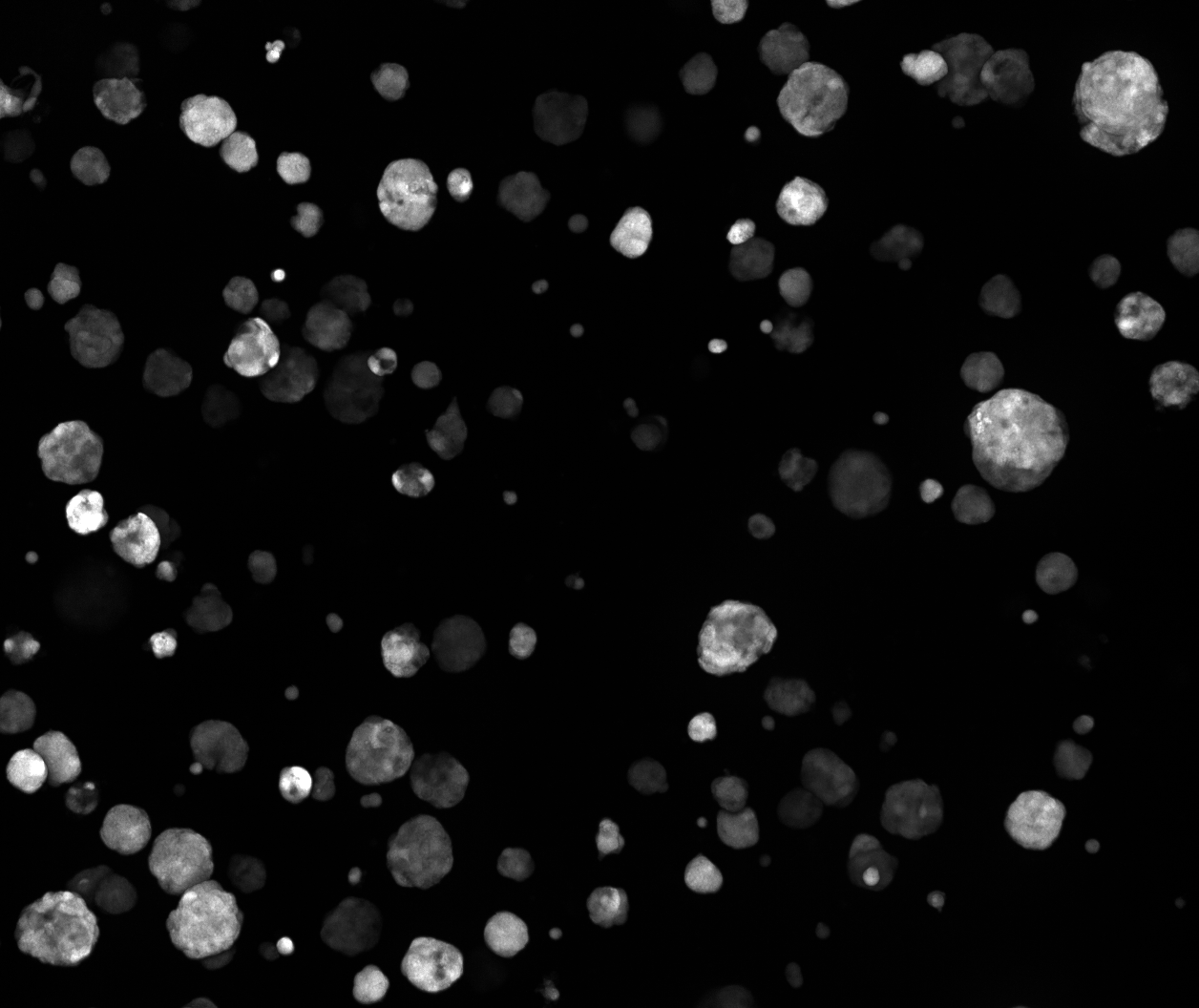

Supplement: S2 File — This is a zip-file containing manually segmented 2D ground truth labeled masks (the corresponding 3D image stacks can be found in the file Data_4 which is available from Dryad (doi:10.5061/dryad.0m9n7)). The cancer (LNCaP) spheroids are labeled in five distinct classes: (1) well separated, (2) overlapping with brighter spheroids in the MIP (rendering it non-separable), (3) overlapping with less bright spheroids in the MIP (rendering it well separable), (4) merely touching other spheroids, and (5) touching the border of the 2D projection of the image. Opening of the images in FIJI (ImageJ) with the ROI Manager allows visual inspection of the data. (ZIP) [file pone.0156942.s007.zip › RoiImage_0335.tif]

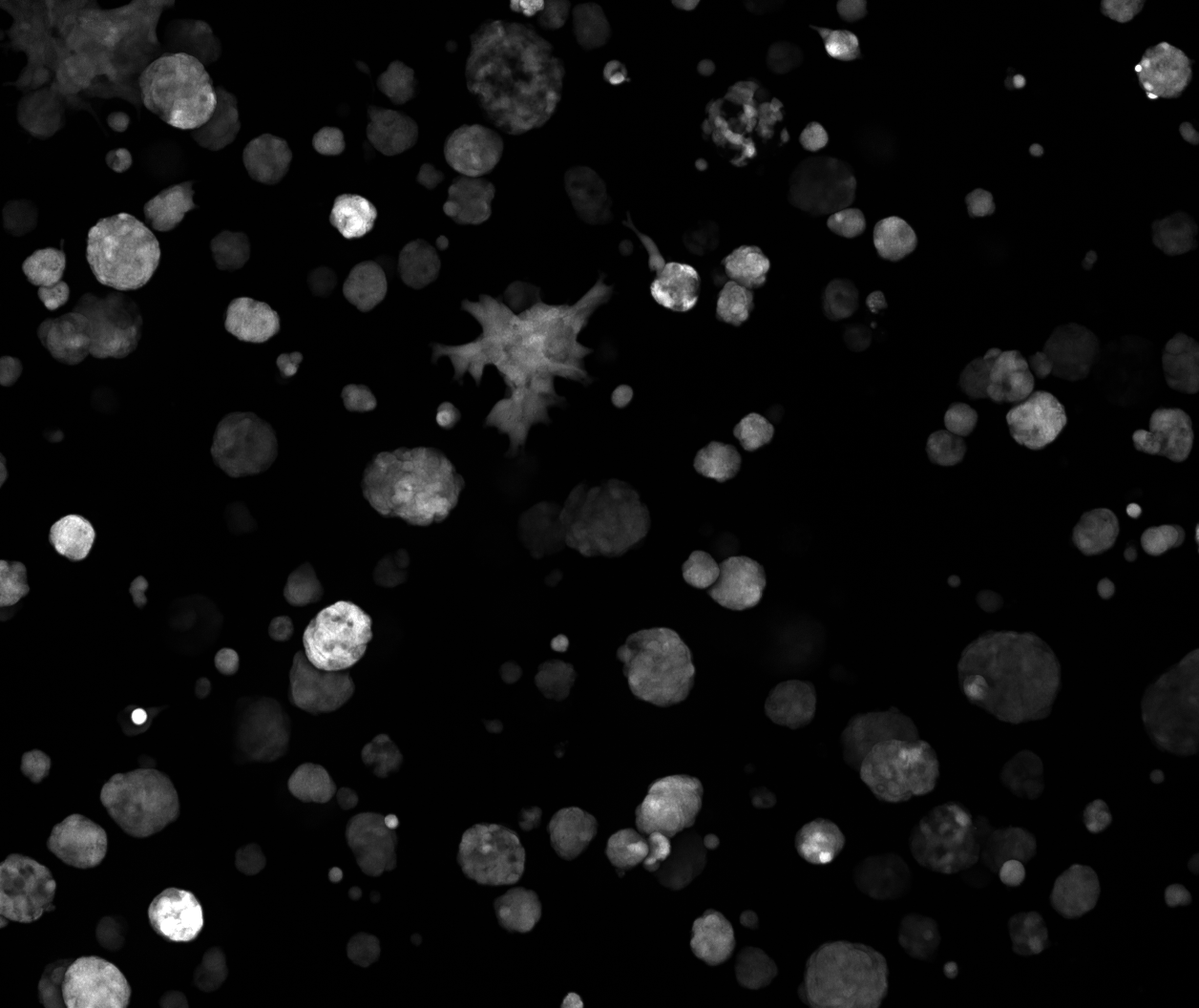

Supplement: S2 File — This is a zip-file containing manually segmented 2D ground truth labeled masks (the corresponding 3D image stacks can be found in the file Data_4 which is available from Dryad (doi:10.5061/dryad.0m9n7)). The cancer (LNCaP) spheroids are labeled in five distinct classes: (1) well separated, (2) overlapping with brighter spheroids in the MIP (rendering it non-separable), (3) overlapping with less bright spheroids in the MIP (rendering it well separable), (4) merely touching other spheroids, and (5) touching the border of the 2D projection of the image. Opening of the images in FIJI (ImageJ) with the ROI Manager allows visual inspection of the data. (ZIP) [file pone.0156942.s007.zip › RoiImage_0336.tif]

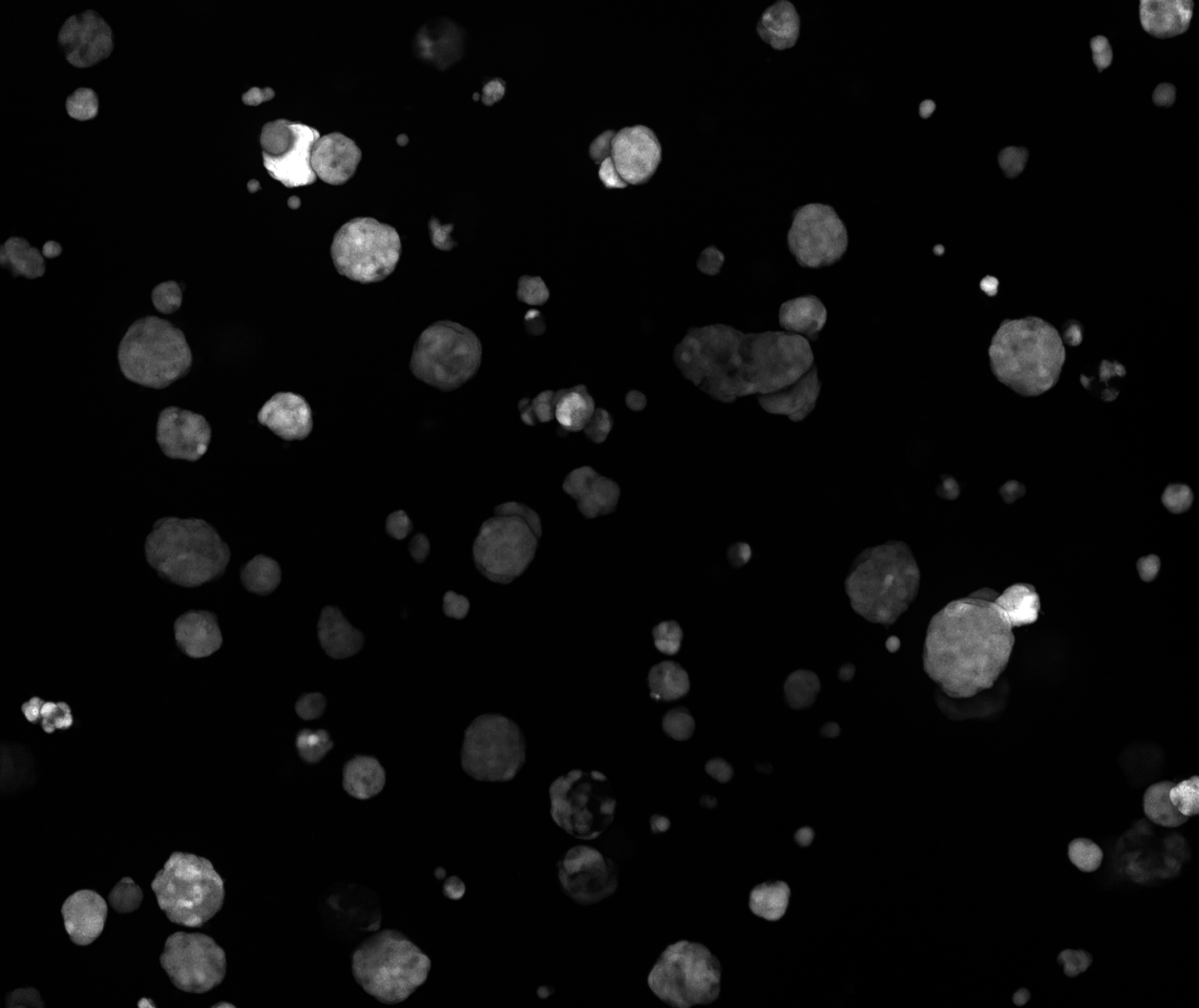

Supplement: S2 File — This is a zip-file containing manually segmented 2D ground truth labeled masks (the corresponding 3D image stacks can be found in the file Data_4 which is available from Dryad (doi:10.5061/dryad.0m9n7)). The cancer (LNCaP) spheroids are labeled in five distinct classes: (1) well separated, (2) overlapping with brighter spheroids in the MIP (rendering it non-separable), (3) overlapping with less bright spheroids in the MIP (rendering it well separable), (4) merely touching other spheroids, and (5) touching the border of the 2D projection of the image. Opening of the images in FIJI (ImageJ) with the ROI Manager allows visual inspection of the data. (ZIP) [file pone.0156942.s007.zip › RoiImage_0337.tif]

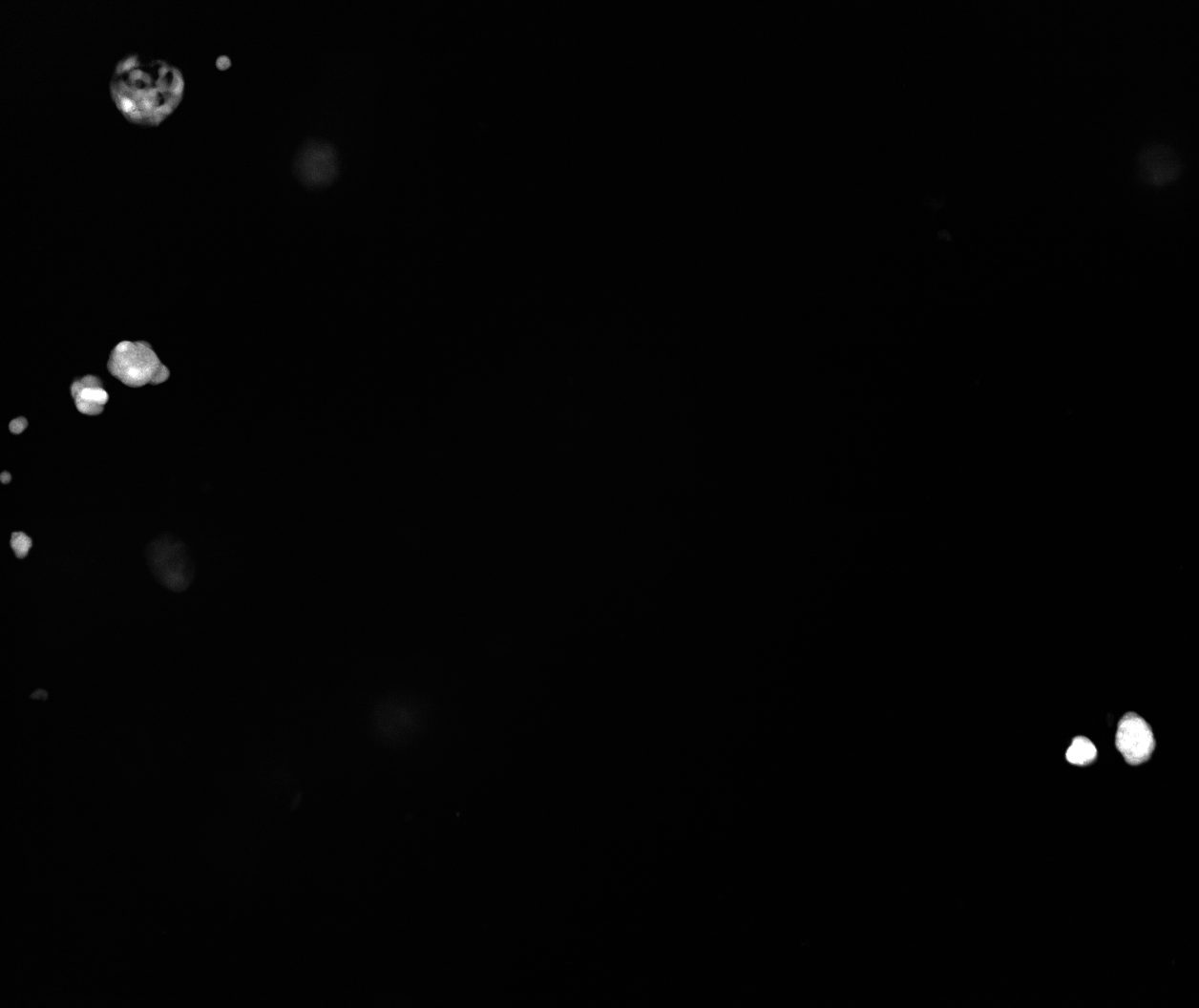

Supplement: S2 File — This is a zip-file containing manually segmented 2D ground truth labeled masks (the corresponding 3D image stacks can be found in the file Data_4 which is available from Dryad (doi:10.5061/dryad.0m9n7)). The cancer (LNCaP) spheroids are labeled in five distinct classes: (1) well separated, (2) overlapping with brighter spheroids in the MIP (rendering it non-separable), (3) overlapping with less bright spheroids in the MIP (rendering it well separable), (4) merely touching other spheroids, and (5) touching the border of the 2D projection of the image. Opening of the images in FIJI (ImageJ) with the ROI Manager allows visual inspection of the data. (ZIP) [file pone.0156942.s007.zip › RoiImage_0338.tif]

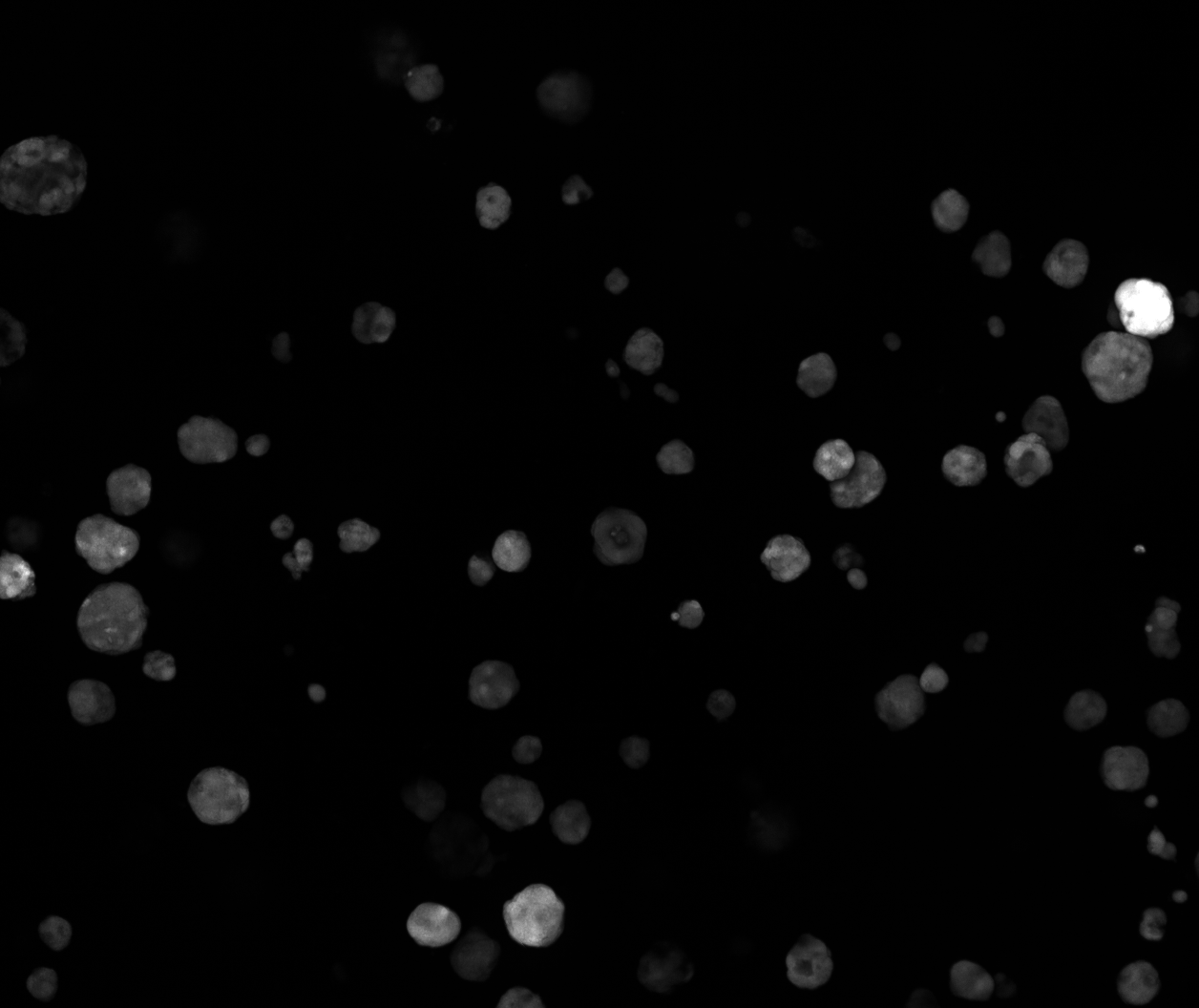

Supplement: S2 File — This is a zip-file containing manually segmented 2D ground truth labeled masks (the corresponding 3D image stacks can be found in the file Data_4 which is available from Dryad (doi:10.5061/dryad.0m9n7)). The cancer (LNCaP) spheroids are labeled in five distinct classes: (1) well separated, (2) overlapping with brighter spheroids in the MIP (rendering it non-separable), (3) overlapping with less bright spheroids in the MIP (rendering it well separable), (4) merely touching other spheroids, and (5) touching the border of the 2D projection of the image. Opening of the images in FIJI (ImageJ) with the ROI Manager allows visual inspection of the data. (ZIP) [file pone.0156942.s007.zip › RoiImage_0339.tif]

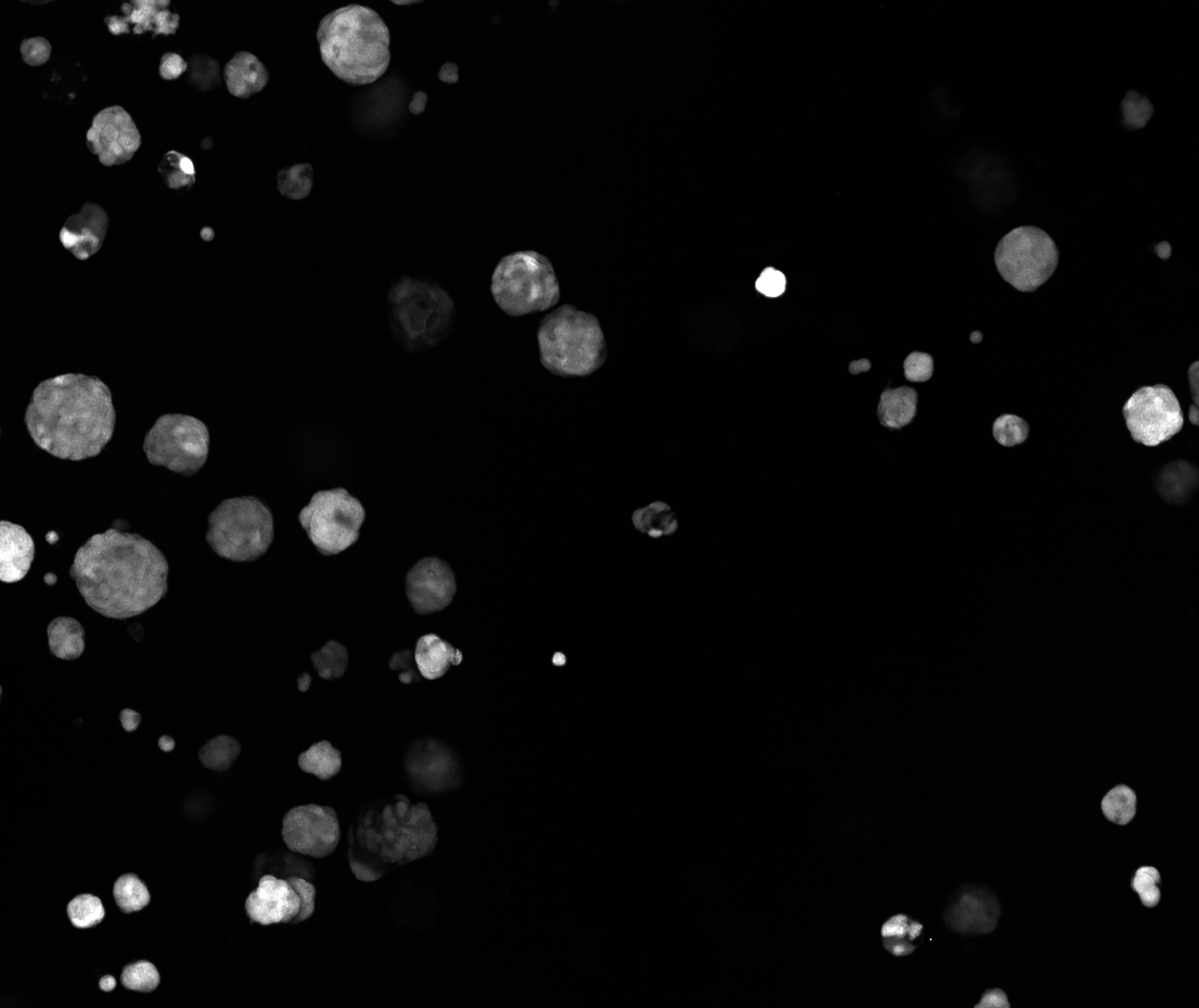

Supplement: S2 File — This is a zip-file containing manually segmented 2D ground truth labeled masks (the corresponding 3D image stacks can be found in the file Data_4 which is available from Dryad (doi:10.5061/dryad.0m9n7)). The cancer (LNCaP) spheroids are labeled in five distinct classes: (1) well separated, (2) overlapping with brighter spheroids in the MIP (rendering it non-separable), (3) overlapping with less bright spheroids in the MIP (rendering it well separable), (4) merely touching other spheroids, and (5) touching the border of the 2D projection of the image. Opening of the images in FIJI (ImageJ) with the ROI Manager allows visual inspection of the data. (ZIP) [file pone.0156942.s007.zip › RoiImage_0340.tif]
